# Production, Consumption, and Potential Public Health Impact of Low- and No-Alcohol Products: Results of a Scoping Review

**DOI:** 10.3390/nu13093153

**Published:** 2021-09-10

**Authors:** Peter Anderson, Daša Kokole, Eva Jané Llopis

**Affiliations:** 1 Department of Health Promotion, CAPHRI Care and Public Health Research Institute, Maastricht University, 6200 MD Maastricht, The Netherlands; d.kokole@maastrichtuniversity.nl (D.K.); eva.jane@esade.edu (E.J.L.); 2 Population Health Sciences Institute, Newcastle University, Newcastle upon Tyne NE2 4AX, UK; 3 ESADE Business School, University Ramon Llull, 08034 Barcelona, Spain; 4 Institute for Mental Health Policy Research, Centre for Addiction and Mental Health (CAMH), Toronto, ON M5S 2S1, Canada

**Keywords:** no-alcohol products, low-alcohol products, production, consumption, health impact

## Abstract

Switching from higher strength to low- and no-alcohol products could result in consumers buying and drinking fewer grams of ethanol. We undertook a scoping review with systematic searches of English language publications between 1 January 2010 and 17 January 2021 using PubMed and Web of Science, covering production, consumption, and policy drivers related to low- and no-alcohol products. Seventy publications were included in our review. We found no publications comparing a life cycle assessment of health and environmental impacts between alcohol-free and regular-strength products. Three publications of low- and no-alcohol beers found only limited penetration of sales compared with higher strength beers. Two publications from only one jurisdiction (Great Britain) suggested that sales of no- and low-alcohol beers replaced rather than added to sales of higher strength beers. Eight publications indicated that taste, prior experiences, brand, health and wellbeing issues, price differentials, and overall decreases in the social stigma associated with drinking alcohol-free beverages were drivers of the purchase and consumption of low- and no-alcohol beers and wines. Three papers indicated confusion amongst consumers with respect to the labelling of low- and no-alcohol products. One paper indicated that the introduction of a minimum unit price in both Scotland and Wales favoured shifts in purchases from higher- to lower-strength beers. The evidence base for the potential beneficial health impact of low- and no-alcohol products is very limited and needs considerable expansion. At present, the evidence base could be considered inadequate to inform policy.

## 1. Introduction

Ethanol in alcoholic beverages is toxic to human health. Whilst consumption of up to 30 g of ethanol a day may be associated with a reduced risk of ischemic heart disease compared with no consumption [1], ethanol is genotoxic and a carcinogen, with no level of risk-free consumption [2].

Alcohol is a risk factor for early death. At an individual level, forty-year-olds who drink more than 350 g of alcohol per week (about five drinks a day) lose four to five years of life compared with those who drink 100 g of alcohol or less per week (approximately one and a half drinks a day) [3]. At a global level, alcohol is the cause of approximately 3 million deaths each year [4].

Reducing alcohol consumption reduces the risk of dying prematurely and the likelihood of a wide range of conditions, including cancer, elevated blood pressure, stroke, liver disease, mental health disorders, and accidents and injuries [5].

There are many strategies that enable people to drink less alcohol. For example, the WHO SAFER initiative calls on governments at all levels to (i) strengthen restrictions on alcohol availability; (ii) advance and enforce drinking and driving countermeasures; (iii) facilitate access to screening, brief interventions, and treatment; (iv) enforce bans or comprehensive restrictions on alcohol advertising, sponsorship, and promotion; and (v) raise prices on alcohol through excise taxes and pricing policies [6].

As an additional strategy, there is a growing discourse around the potential public health benefit of low- and no-alcohol products (alcohol-free and low-alcohol versions of alcoholic drinks such as beer, wine, and spirits) [7,8]. The WHO’s global alcohol strategy called on the alcohol industry to contribute to reducing the harmful use of alcohol by addressing its products [9], for example, by reducing the amount of alcohol they contain. In its consultation document, ‘Advancing our health: prevention in the 2020s,’ the UK Government made a commitment to work with the alcohol industry to deliver a significant increase in the availability of alcohol-free and low-alcohol products by 2025; [10].

Low- and no-alcohol products can only be of public health benefit if they replace rather than add to existing consumption of higher strength products. In addition, any such potential health benefits resulting from the production and replacement consumption of low- and no-alcohol products should be offset against any environmental external costs (for example, due to extra steps in production), which can be assessed through life cycle assessments.

To inform the discourse on low- and no-alcohol products, we have undertaken a scoping review on their production, consumption, and potential health impact. In the review, we identify five research questions, for which we aim to synthesise knowledge about low- and no-alcohol products related to:

1. Production, including life cycle assessment compared with production of regular strength products
2. Prevalence of purchase and consumption
3. Potential health impact
4. Consumer perceptions and preferences
5. Policy drivers of purchase and consumption.

## 2. Methods

### 2.1. Design and Registration

We used scoping review methodology because of the breadth of the research questions and the lack of clarity on the amount and nature of existing research on this topic. Scoping reviews are used to map the main concepts in research areas and present a broad overview of the existing evidence, including the identification of research gaps, regardless of the study quality [11]. The design was guided by the methodological framework of Arksey and O’Malley [12]. The review protocol was pre-registered at https://osf.io/kv3rj/ (accessed on 1 September 2021).

### 2.2. Eligibility Criteria

To be included in the review, papers had to include topics related to the production, consumption, and health impact of low- and no-alcohol products. There was no restriction on research design. To delineate the scope of our research, we only focused on peer-reviewed literature rather than grey literature. Original articles and reviews in English published in 2011 or later were included. For production, only reviews were included, as a detailed examination of production methods was not the focus of the paper. Papers not specific to low- and no-alcohol products were excluded.

### 2.3. Information Sources and Search Strategy

Two databases (PubMed and Web of Science) were searched with the abovementioned restrictions on language and dates (English language, published between 1 January 2011 and 17 January 2021). The search strategy contained blocks with terms related to low- and no- alcoholic products (‘Low alcohol’ or ‘No alcohol’ or ‘Zero alcohol’ or ‘Alcohol-free’ or ‘Alcohol free’ or Reformulation or Reduc* ethanol content or Reduc* ethanol strength or Reduc* ‘alcohol strength’ or Reduc* ‘alcohol content’ or Low strength alcohol OR ‘non-alcoholic’) and (beer or cider or wine or spirits or ready to drink or fortified wine or fermented beverages or intermediate products) in combination with other blocks related to production, consumption, and impact, producing several searches (see Tables S1 and S2 for a description of blocks and full search strategy). Database searches were complemented with Google Scholar inspection and reference searches.

### 2.4. Study Selection and Summary 

Study selection was performed by two researchers (DK and PA) on the basis of the abovementioned inclusion and exclusion criteria. After the removal of duplicates, the studies were first screened by title and abstract by one researcher, followed by full-text examination and final article selection. Any doubts were discussed and resolved by consensus. The selected articles were grouped according to their main themes. A data extraction form was prepared to collect information on the authors, year of publication, paper objectives, studied topic, and key findings or conclusions. No quality appraisal of studies was undertaken, as the purpose of the review was to map all the available literature. For each theme, the main findings are presented in a narrative manner.

## 3. Results

**Figure 1:**
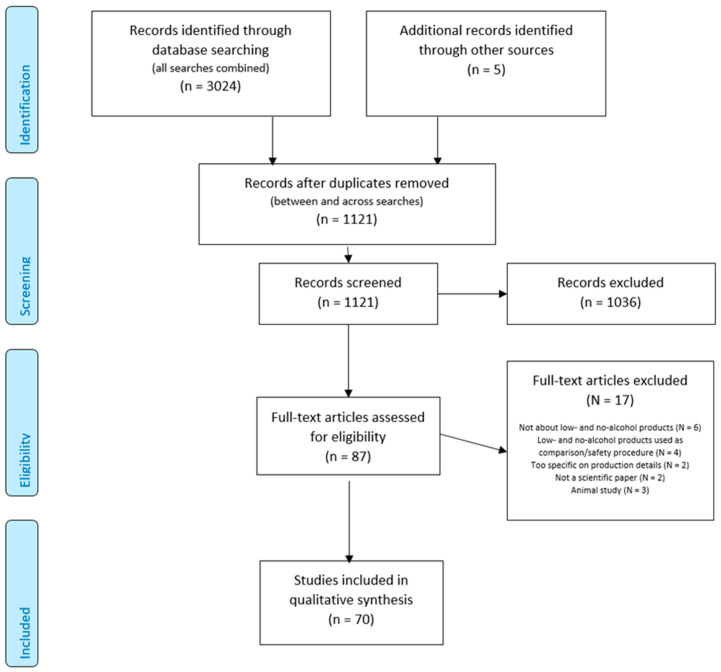
PRISMA flow diagram.

In total, 3024 papers were identified across the eight conducted searches, and five papers were identified from other sources (Figure 1). After the removal of duplicates across the two databases and between the searches, 1121 papers remained for title and abstract screening. Ninety papers were selected for full-text inspection, and 70 of them were selected for final qualitative synthesis (see Table S3).

We matched the thematic analysis of each paper’s topic to the five main research questions: production and life-cycle assessment, consumption and purchase, the potential impact on health, perception and preferences, and policy drivers of purchase and consumption. The results are presented within these categories. Additionally, several reviews were found which encompassed broader overviews of the selected topics and sometimes covered several themes at once [13,14,15,16,17,18,19,20]; thus, their findings are presented where appropriate. Along with the key findings of the papers, we also examined the definition of low- and no-alcohol products and the funding sources for the research (see Table S4). 

### 3.1. Production and Life Cycle Assessment

In terms of the production of low- and non-alcoholic beverages, the majority of the research focused on beer and wine. For non-alcoholic or low-alcohol beer, the reason given for the importance of improving production used in many papers is flavour is one of the main problems with non-alcoholic beer acceptance. Thus, much of the production research has focused on how to make the flavour more acceptable and similar to alcoholic beer and whether biological (limiting ethanol formation during beer fermentation) or physical (removing ethanol from regular beer) approaches are more suitable to achieve this goal [21,22,23,24,25,26,27]. Most of the reviews refer to regulations when defining beer products; for example, EU regulations require 0.5% alcohol by volume (ABV) or less for non-alcoholic beer and 1.2% ABV or less for low-alcohol beer, but they acknowledge that these regulations differ by country. Other directions of research are related to low alcoholic or alcohol-free beers within the niche of craft beer [13] and dry hopping as a technique to produce non-alcoholic beer [14].

In the field of wine, the de-alcoholisation research has two directions: one objective is to decrease the alcohol strength in wine, and the second is to produce new low-alcohol beverages [28]. With regard to the first objective, the main issue that the wine industry faces is the increase in wine ABV due to climate change conditions (e.g., in Australian red wines, alcohol concentration has increased approximately 1% ABV per decade since the 1980s [29]). Thus, research has focused on how to reduce the elevated alcohol content in wine without leading to a taste that is not as well-accepted by consumers (e.g., [29,30,31]). For the second objective, the issue is also to overcome the loss of desirable sensory properties appreciated by consumers when alcohol content is reduced/removed from wine, with research focusing on identifying the most appropriate methods to achieve this goal (e.g., [32,33,34,35]). In terms of the definition of lower-alcohol wine products, the reviews have acknowledged country differences in regulations: one review mentioned the definitions adopted by the International Organisation of Vine and Wine: ’Beverages obtained by wine dealcoholization’ for beverages with 0.5% ABV or lower, and ‘Beverages obtained by partial wine dealcoholization’ for those in the range of 0.5–8.5% ABV [28]; other reviews have defined wine products as lower-alcohol with an ABV of up to 11.0% [33,34].

While the comparison of various production methods is difficult, and thus it is difficult to define the best processes [22], some attempts at life cycle assessment have been carried out for alcohol-free bitter extracts as aperitifs [36,37] as well as for partial de-alcoholisation of wines [38]. We found no published life cycle assessments comparing the production of zero-alcohol beers or wines with the production of regular beers or wines.

As mentioned previously, no research was found on alcohol-free or low-alcohol spirits, but another review examined the field of traditional low-alcoholic and non-alcoholic fermented beverages [39], such as kefir, boz, or kvass. Finally, we found one study of ethanol production in non-alcoholic beer across the storage period, which found that storage temperature and packaging can have significant effects on ethanol production during the storage period, although the excess production does not exceed the allowed amount of 0.5% ABV [40].

### 3.2. Consumption and Purchase 

Relatively little research in peer-reviewed literature has focused on low- and no-alcohol drink consumption and purchase trends, and all studies have focused on non-alcoholic or low-alcohol beer (none on wine or spirits). One study [41,42] examined the introduction of new low- and no-alcohol beers (defined as beers with 3.5% ABV or less) and reformulated beers in Great Britain and found that the volume of purchases of new low- and no-alcohol beer products (2.6% of the volume of all beers purchased in 2018) and new reformulated beer products (6.9% of the volume of all beers purchased in 2018) was very small. More widely, an analysis of official data from ethanol beer sales in Australia and New Zealand [43] showed that in Australia, the consumption of ethanol in mid-strength beer (3.01–3.5% ABV) increased, whereas consumption of low-strength beer (<3% ABV) decreased between 2000 and 2016. In New Zealand, the consumption of mid-strength beer (2.501–4.35% ABV) decreased substantially.

### 3.3. Impact on Health

Overall, only one public health-oriented review has been conducted to examine the evidence base of the reduction of ethanol content of alcoholic beverages as a means to reduce the harmful use of alcohol [20]. The review concluded that the literature is still too scarce to draw conclusions, although some mechanisms for how this might occur have been proposed: first, current drinkers may replace standard alcoholic beverages with similar beverages of lower alcoholic strength, and second, current drinkers may switch to no-alcohol alternatives some of the time. On the other hand, lowering alcoholic strength could reduce the threshold and initiate alcohol use in current abstainers, especially in adolescents.

The study [41] that examined the impact of the introduction of new low- and no-alcohol beers (defined as beers with 3.5% ABV or less) and reformulated beers in Great Britain on the average alcoholic strength of beer and the number of grams of alcohol purchased by households obtained the following results: a combined associated impact of both events with relative reductions of alcohol by volume of beer between 1.2% and 2.3%, purchases of grams of alcohol within beer between 7.1% and 10.2%, and purchases of grams of alcohol as a whole between 2.6% and 3.9%. Another study investigated the reformulation of products by one company and found that the mean ABV of its beer products dropped from 4.69 in 2015 to 4.55 in 2018, and these changes were associated with reduced purchases of grams of alcohol within its beer products [42].

Several individual studies have focused on the impact of low- and no-alcohol products on health-related topics. All but one focused on beer. The topics covered have included the impact of non-alcoholic beer on anxiety [44] and sleep quality [45,46]; the impact of alcohol-free beer enriched with isomaltulose on insulin resistance in diabetic patients with overweight or obesity [47]; the effect of non-alcoholic beer compared with improved diet and exercise on nutritional status, endothelial function, and quality of life in patients with cirrhosis [48]; the impact of non-alcoholic beer [49,50,51] or alcohol-free wine [52] on cardiovascular health; and the relationship between non-alcoholic beer and breastfeeding in terms of whether supplementing with non-alcoholic beer improves the oxidative stress and antioxidant content of breast milk [53] as well as how much ethanol in non-alcoholic beer may reach the breastfed child [54]. All these studies concluded that the impact of the tested drink was in a favourable direction, but they focused on relatively short-term effects (days to months) and the studies were conducted on small samples (ranging between 7 and 60 participants). The only study examining effects over a longer term (two years) found that in Australian older women, the frequency of drinking low-alcohol beer was positively associated with bone mass density in the lumbar spine but not in the hip [55]. Additionally, among the studies mentioned in this section, only two of twelve [52,55] were not funded by the alcohol industry or industry-related organisations. The majority of the studies also did not specifically define the possible alcohol content in the drinks, although they used terms in line with those used in regulations (alcohol-free, non-alcoholic, and low-alcohol). Additionally, two reviews examined the health properties of low-alcohol and alcohol-free beer [15,16] but focused more on the theorised nutritional benefits of beer and how to retain them in low- and non-alcoholic alternatives.

Another prominent topic in terms of study focus is the evaluation of non-alcoholic or low-alcohol beer in the context of sports/exercise, studying these products’ effect on decreasing post-race inflammation and upper respiratory tract infection incidence among marathon runners [56] or fluid retention after exercise, with mixed results [57,58,59,60]. Finally, one study investigate chemical and physical properties to determine whether beer can be considered an isotonic drink [61] and concluded that only yeast-clouded alcohol-free beer (but not regular beer or clear alcohol-free beer) could be declared and promoted as isotonic, as it matches the Codex Alimentarius threshold values. However, none of the tested beverages matched the EC recommendation for sodium content. 

### 3.4. Perceptions and Preferences

A relatively large number of papers focused on examining individual perceptions and preferences related to low- and non-alcoholic beverages, although the range of examined topics was rather heterogeneous. The majority of studies focused on beers, although regarding health impact, more studies focused on low-alcohol wine, and one considered alcohol-free spirits.

Several studies examined responses to low- and non-alcoholic beverages (beer and wine). One study found that, while the alcohol content affected participants’ sensory expectations, it had no significant effect on expected liking; it also found that describing the sensory qualities of beer using a sensory descriptor had a larger effect than labelled alcohol content and label colour [62]. Another study [63] investigated whether non-alcoholic beer induced a conditioned response even when participants know that the beer is non-alcoholic. The study found that non-alcoholic beer produced a conditioned response in older drinkers because these drinkers more strongly associated the alcohol conditioned stimulus with the unconditioned stimulus than did younger participants [63]. One study [64] investigated how people evaluate low-alcohol wine (8% ABV content) and if the reduction in alcohol and the information that a wine is low in alcohol influenced consumption. The study found no difference in liking and consumption between low-alcohol and standard-alcohol wines [64]; however, participants were willing to pay more for standard wine compared with lower-alcohol wine. Two studies used functional magnetic resonance approaches to compare peoples’ reactions to lower-alcohol alternatives and higher-alcohol alternatives: one [65] found no differences between acute brain rewards in the consumption of beer with and without alcohol when presented in a context in which regular alcoholic beer is expected; the second study [66] found greater activation in brain regions that are sensitive to taste intensity in low-alcohol compared with high-alcohol wines, although the definition of low-alcohol wine in the study was 13–13.5% ABV. Two studies examined whether consumers were able to discriminate between alcoholic and non-alcoholic products; both found that consumers were able to distinguish between mock and real sparkling wine [67] and between alcohol-free spirits and alcoholic spirits [68].

A number of studies investigated preferences and found that taste is an important preference driver in Australian wine consumers [69] and that that innovative wine attributes, including alcohol-free wine, were ranked among the least important attributes in a sample of Italian wine consumers [70]. Alcoholic aroma and flavour contributed as positive preference drivers for the acceptance of non-alcoholic beer in a sample of beer consumers [71]. Consumers perceived light beer (not clearly defined by % ABV) as healthier but less tasty than regular beer, and the preference for light beer was driven mostly by taste, prior experience, and brand [72]. A study of regular beer or wine consumers from the UK found that participants perceived pregnant women, athletes, and those aged 6–13 years old as target groups, and they perceived weekday lunches as the target occasions for drinking wine and beer labelled as lower-strength [73]. Another survey of Australian wine consumers found that taste was an important driver of consumption and considered ‘low-alcohol wine’ to contain around 3–8% alcohol [69]. The perceived reasons for preferring a low-alcohol wine included driving after drinking, lessening the adverse effects of alcohol, and being able to consume more without the effects of higher-alcohol wine [69]. Finally, in a sample of Dutch and Portuguese respondents, non-alcoholic beer was conceptualized as useful when alcohol was not convenient and functional as a substitute for regular beer, with the consumption of non-alcoholic beer driven by health and wellbeing issues, price differentials, and overall decreases in the social stigma associated with drinking alcohol-free beverages [74,75].

It is important to note that perceptions and preferences of low- and no-alcohol products might differ by culture and country. Two reviews examined the cultural context of non-alcoholic or low-alcohol beer consumption in the US [19] and the Netherlands and Portugal [17]. Another review pointed out that much low alcohol wine research originates from Australia [18].

### 3.5. Policy Drivers

We identified a number of policy driver-related studies, such as marketing [76] and labelling of lower-strength beer and wine [77,78,79] as well as the price of low- and non-alcoholic beer [80,81,82], with funding either received by governmental bodies or not received at all. In terms of marketing, one study conducted a content analysis of how the low- or lower-strength equivalents of beer and wine were marketed in an online context in the UK [76] and found that they were more often marketed in association with occasions deemed to be suitable for their consumption, including lunchtime (for wine), outdoor events/barbeques (for beer), and on sport/fitness occasions (for beer). Compared with regular-strength wines and beers, low- and lower-strength equivalents were more frequently marketed with images or text associated with health or information about low alcohol content and appeared to be marketed not as substitutes for higher-strength products but as products that can be consumed on additional occasions with an added implication of healthiness.

Two studies considered the labelling of low- and non-alcoholic products among regular drinkers from the UK. One study found that products with verbal descriptors denoting lower strength (low and super-low) had a lower appeal than regular-strength products, with appeal decreasing as % ABV decreased [79]. The second study found that the total amount of drink consumed increased as the label on the drink denoted successively lower alcohol strengths [78]. A related study also found that 17 of 18 verbal descriptors for lower-strength products were perceived as denoting products far higher in strength than the currently legislated cap (in the UK) of 1.2% ABV for low-alcohol products [77].

Related to price, one study [80] found that the introduction of minimum unit price in Scotland and Wales shifted purchases from higher- to lower-strength products, more so for ciders than for beers. In Australia, one analysis [82] found that after the varying nominal rates of tax were introduced for beer products according to three alcohol content levels (low-, mid-, and high-strength) in 2000/01, the relatively higher nominal tax rates for two beer categories (mid- and high-strength off-premises) had a significant negative effect on their consumption. Another Australian study examined price elasticity for several alcoholic beverages, including low-alcohol beer, but did not report on this specific category [81].

## 4. Discussion

The main finding of this scoping review was that there is only a relatively small and incoherent scientific literature on the production, consumption, and potential health impact of low- and no-alcohol products. The evidence base could be considered insufficient to inform policy.

Producers have a responsibility to report on their health and environmental impacts. Despite European guidance on undertaking life cycle assessments for beer production [83], such assessments are voluntary, with no requirement for public reporting. Whilst alcohol-free products, for example, may have a potential impact in reducing the harm inflicted by alcohol, and such potential benefits should be weighed against any environmental external costs, which may be present because there are extra steps in the production processes. Such scientific assessments have not been published.

There are hardly any published scientific data on the extent of purchase and consumption of low- and no-alcohol products. Published British data, which is restricted to household purchases of beer, suggest only low penetration in terms of volumes purchased.

In terms of health impact, there are a number of disparate studies on potential low quality, from which it is difficult to draw firm conclusions. British household purchase data suggest that the introduction of new low- and no-alcohol beers as well as product reformulation of existing beers to contain less alcohol result in households purchasing fewer grams of ethanol. However, these analyses refer to only one product (beer) and only one jurisdiction (Great Britain).

There have been several studies on perceptions and preferences for the uptake of no- and low-alcohol products, primarily beer. These indicate the importance of taste, prior experiences, brand (same brand as regular-strength beers), health and wellbeing issues, price differentials, and overall decreases in the social stigma associated with drinking alcohol-free beverages as important drivers of the purchase and consumption of low- and no-alcohol beers.

With respect to policy drivers, some research suggests that low- and no-alcohol products are marketed as products that can be used on additional occasions or in additional circumstances rather than as substitutes for higher-strength products. Although concern has been expressed concern on this issue [7], the scoping review found no published studies to indicate whether or not the marketing of low- and no-alcohol brands is specifically used to market higher-strength products. Several studies on labelling have suggested that the labelling of low- and no-alcohol products is sometimes inconsistent and not always as clear as it should be. Finally, some evidence from several jurisdictions suggests that pricing policy, such as the introduction of a minimum unit price, can favour shifts in purchases and consumption from higher- to lower-strength alcohol products.

### 4.1. Limitations

Our review is subject to the general limitations of scoping reviews: we did not appraise studies for quality, and the conclusions are still somewhat broad and qualitative. The results of our review are constrained by the relatively small size of published studies, which were not necessarily coherent within the five main groups that we used. In addition, as we started with very broad research questions, we decided to limit certain methodological criteria (such as using only two search databases and focusing on peer-reviewed articles in English only) in order to rapidly find and appraise the most relevant literature, but this means that findings from grey literature in other languages (such as government reports) are not included in this review.

### 4.2. Implications

Despite its limitations, our review indicates large research gaps and identifies policy actions to address these gaps, as proposed in the Table 1.

## 5. Conclusions

At present, the published scientific literature on low- and no-alcohol products is too scarce and incoherent to adequately inform policy. Although analyses from one jurisdiction (Great Britain) suggest that at least low- and no-alcohol beers might be associated with reduced purchases of ethanol overall, there needs to be a rapid extension of published research across different jurisdictions and different product categories. Such research requires a series of natural experiments on new product development and availability, along with new policy drivers that promote shifts from higher- to lower-strength products that are fully and independently evaluated for their impact on drinking less ethanol, thus reducing ill-health and premature death. Such research should be a required price tag for all relevant natural experiments led by producers and governments.

## Figures and Tables

**Table 1:** Research and policy implications.

Future Research
There needs to be a major investment and expansion in scientific research on low and no alcohol products that covers, at least, the following topic areas: ○ Production: Full life cycle assessments comparing the health and environmental impacts of the production of low- and no-alcohol products compared with the production of the same or similar branded regular strength products ○ Purchase and Consumption: Detailed analyses of the purchase and consumption of low and no-alcohol products across different product categories (e.g., beers, wines, and spirits) in a range of jurisdictions and over time. Detailed analyses of the socio-demographic characteristics of who buys and drinks low- and no-alcohol products in terms of gender, age, income, educational level, occupational group, index of residential deprivation, and geographical area ○ Perceptions and Preferences: at the individual level, what drives the purchase and consumption of low- and no-alcohol products, including drinking occasion and location; taste, health, and well-being concerns; and previous experiences and loyalty to brands (of regular strength products) ○ Health Impact: Does the introduction of new low- and no-alcohol products and the new purchase and consumption of such products result in consumers drinking fewer grams of alcohol (sustainable amounts) over time? If less ethanol is purchased and consumed, how does this differ by socioeconomic characteristics of consumers? ○ Policy Drivers: From an evidence perspective, what are the most appropriate definitions of low- and no-alcohol products across different categories (e.g., beers, wines, and spirits)? How should low- and no-alcohol products be labelled to adequately and accurately inform consumers? How should low- and no-alcohol products be placed in stores to best promote their purchase at the expense of higher strength products? How should the marketing of low and no-alcohol products be regulated to prevent any negative impact of marketing in leading to increased consumption of ethanol? To what extent can pricing policy, including a minimum unit price, encourage the purchase of low- and no-alcohol products at the expense of higher strength products.
**Policy Implications**
○ Clear standards and definitions need to be put in place regarding the definitions of low- and no-alcohol products across the different categories of beers, wines, and spirits, recognising, for example, that low-alcohol equivalents of spirits cannot be currently classified as spirits. ○ On one hand, there is a dearth of scientific information to adequately inform policy ○ On the other hand, there is a need for natural policy experiments in which low- and no-alcohol products are produced, marketed, and supported by relevant policy in the domains of marketing requirements, labelling, and pricing ○ The proviso is that all initiatives and natural policy experiments are fully and adequately subject to independent evaluation ○ Published life cycle assessments should become a mandatory requirement of all alcohol product development, building on existing guidance, and specifically comparing the production of no-alcohol products with the same branded regular strength products

## Data Availability

Extracted data is presented in the Supplementary Materials.

## Supplementary Materials

The following are available online at https://www.mdpi.com/article/10.3390/nu13093153/s1.

## References

[1] Rehm J., Rovira P., Shield K.D. (2021). Dose-response relationships between levels of alcohol use and risks of mortality for all people, and for different ages, by sex, and for groups defined by risk factors. Nutrients.

[2] Rumgay H., Murphy N., Ferrari P., Soerjomataram I. (2021). Alcohol and cancer: Epidemiology and biological mechanisms. Nutrients.

[3] Wood A., Kaptoge S., Butterworth A., Paul D., Burgess S., Sweeting M., Bell S., Astle W., Willeit P., Bolton T. (2018). Risk thresholds for alcohol consumption: Combined analysis of individual-participant data for 599,912 current drinkers in 83 prospective studies. Lancet.

[4] Shield K., Manthey J., Rylett M., Probst C., Wettlaufer A., Parry C.D.H., Rehm J. (2020). National, regional, and global burdens of disease from 2000 to 2016 attributable to alcohol use: A comparative risk assessment study. Lancet Public Health.

[5] World Health Organization (WHO) (2018). Global Status Report on Alcohol and Health. https://apps.who.int/iris/bitstream/handle/10665/274603/9789241565639-eng.pdf?ua=1&ua=1.

[6] World Health Organization (2020). SAFER, Alcohol Control Initiative. https://www.who.int/substance_abuse/safer/en/.

[7] European Health and Digital Executive Agency Service Contract for the Provision of 2019—Support to Member States in Studies and Capacity Building Activities to Reduce Alcohol Related Harm. https://etendering.ted.europa.eu/cft/cft-document.html?docId=65290.

[8] Corfe S., Hyde R., Shepherd J. Alcohol-Free and Low-Strength Drinks Understanding Their Role in Reducing Alcohol-Related Harms. https://www.smf.co.uk/publications/no-low-alcohol-harms/.

[9] World Health Organization (2010). Global Strategy to Reduce the Harmful Use of Alcohol.

[10] UK Government (2019). Advancing our Health: Prevention in the 2020s—Consultation Document. https://www.gov.uk/government/consultations/advancing-our-health-prevention-in-the-2020s/advancing-our-health-prevention-in-the-2020s-consultation-document.

[11] Arksey H., O’Malley L. (2005). Scoping studies: Towards a methodological framework. Int. J. Soc. Res. Methodol..

[12] Tricco A.C., Lillie E., Zarin W., O’Brien K., Colquhoun H., Kastner M., Levac D., Ng C., Sharpe J.P., Wilson K. (2016). A scoping review on the conduct and reporting of scoping reviews. BMC Med. Res. Methodol..

[13] Baiano A. (2020). Craft beer: An overview. Compr. Rev. Food Sci. Food Saf..

[14] Hagemann M.H., Bogner K., Marchioni E., Braun S. (2016). Chances for dry-hopped non-alcoholic beverages? Part 1: Concept and market prospects. Brew. Sci..

[15] Hagemann M.H., Schmidt-Cotta V., Marchioni E., Braun S. (2017). Chance for Dry-hopped Non-alcoholic Beverages? Part 2: Health Properties and Target Consumers. Brew. Sci..

[16] Mellor D.D., Hanna-Khalil B., Carson R. (2020). A Review of the Potential Health Benefits of Low Alcohol and Alcohol-Free Beer: Effects of Ingredients and Craft Brewing Processes on Potentially Bioactive Metabolites. Beverages.

[17] Silva A.P., Jager G., Van Zyl H., Voss H.-P., Pintado M.M., Hogg T., De Graaf C. (2017). Cheers, proost, saúde: Cultural, contextual and psychological factors of wine and beer consumption in Portugal and in the Netherlands. Crit. Rev. Food Sci. Nutr..

[18] Bucher T., Deroover K., Stockley C. (2018). Low-Alcohol Wine: A Narrative Review on Consumer Perception and Behaviour. Beverages.

[19] Hoalst-Pullen N., Patterson M.W. (2020). The Geography of Beer: Culture and Economics.

[20] Rehm J., Lachenmeier D.W., Llopis E.J., Imtiaz S., Anderson P. (2016). Evidence of reducing ethanol content in beverages to reduce harmful use of alcohol. Lancet Gastroenterol. Hepatol..

[21] Blanco C.A., Andrés-Iglesias C., Montero O. (2016). Low-alcohol Beers: Flavor Compounds, Defects, and Improvement Strategies. Crit. Rev. Food Sci. Nutr..

[22] Brányik T., da Silva D.P., Baszczyňski M., Lehnert R., Silva J.B.D.A.E. (2012). A review of methods of low alcohol and alcohol-free beer production. J. Food Eng..

[23] Catarino M., Mendes A. (2011). Non-alcoholic beer—A new industrial process. Sep. Purif. Technol..

[24] Jackowski M., Trusek A. (2018). Non-alcoholic beer production—An overview. Pol. J. Chem. Technol..

[25] Muller C., Neves L.E., Gomes L., Guimarães M., Ghesti G. (2020). Processes for alcohol-free beer production: A review. Food Sci. Technol..

[26] Mangindaan D., Khoiruddin K., Wenten I.G. (2018). Beverage dealcoholization processes: Past, present, and future. Trends Food Sci. Technol..

[27] Salanta L.C., Coldea T.E., Ignat M.V., Pop C.R., Tofana M., Mudura E., Zhao H.F. (2020). Non-Alcoholic and Craft Beer Pro-duction and Challenges. Processes.

[28] Liguori L., Albanese D., Crescitelli A., Di Matteo M., Russo P. (2019). Impact of dealcoholization on quality properties in white wine at various alcohol content levels. J. Food Sci. Technol..

[29] Varela C., Dry P.R., Kutyna D.R., Francis I.L., Henschke P.A., Curtin C.D., Chambers P.J. (2015). Strategies for reducing alcohol concentration in wine. Aust. J. Grape Wine Res..

[30] Dequin S., Escudier J.-L., Bely M., Noble J., Albertin W., Masneuf-Pomarède I., Marullo P., Salmon J.-M., Sablayrolles J.M. (2017). How to adapt winemaking practices to modified grape composition under climate change conditions. OENO One.

[31] Ozturk B., Anli E. (2014). Different techniques for reducing alcohol levels in wine: A review. BIO Web Conf..

[32] Longo R., Blackman J.W., Torley P.J., Rogiers S.Y., Schmidtke L.M. (2017). Changes in volatile composition and sensory attributes of wines during alcohol content reduction. J. Sci. Food Agric..

[33] Longo R., Blackman J.W., Antalick G., Torley P.J., Rogiers S.Y., Schmidtke L. (2018). A comparative study of partial dealcoholisation versus early harvest: Effects on wine volatile and sensory profiles. Food Chem..

[34] Longo R., Blackman J.W., Antalick G., Torley P.J., Rogiers S.Y., Schmidtke L.M. (2018). Harvesting and blending options for lower alcohol wines: A sensory and chemical investigation. J. Sci. Food Agric..

[35] Varela J., Varela C. (2019). Microbiological strategies to produce beer and wine with reduced ethanol concentration. Curr. Opin. Biotechnol..

[36] Laoretani D.S., Sánchez R.J., Paredes D.A.F., Iribarren O.A., Espinosa J. (2020). On the conceptual modeling, economic analysis and life cycle assessment of partial dealcoholization alternatives of bitter extracts. Sep. Purif. Technol..

[37] Paredes D.A.F., Laoretani D.S., Morero B., Sánchez R., Iribarren O.A., Espinosa J. (2020). Screening of membrane technologies in concentration of bitter extracts with simultaneous alcohol recovery: An approach including both economic and environmental issues. Sep. Purif. Technol..

[38] Margallo M., Aldaco R., Barceló A., Diban N., Ortiz I., Irabien A. (2015). Life cycle assessment of technologies for partial dealcoholisation of wines. Sustain. Prod. Consum..

[39] Baschali A., Tsakalidou E., Kyriacou A., Karavasiloglou N., Matalas A.-L. (2017). Traditional low-alcoholic and non-alcoholic fermented beverages consumed in European countries: A neglected food group. Nutr. Res. Rev..

[40] Zendeboodi F., Jannat B., Sohrabvandi S., Khanniri E., Mortazavian A.M., Khosravi K., Sarmadi B., Asadzadeh S., Amirabadi P.E., Esmaeili S. (2019). Monitoring of ethanol content in non-alcoholic beer stored in different packages under different storage temperatures. Biointerface Res. Appl. Chem..

[41] Anderson P., Llopis E.J., O’Donnell A., Manthey J., Rehm J. (2020). Impact of low and no alcohol beers on purchases of alcohol: Interrupted time series analysis of British household shopping data, 2015–2018. BMJ Open.

[42] Anderson P., Llopis E.J., Rehm J. (2020). Evaluation of Alcohol Industry Action to Reduce the Harmful Use of Alcohol: Case Study from Great Britain. Alcohol Alcohol..

[43] Kypri K., Harrison S., McCambridge J. (2020). Ethanol Content in Australian and New Zealand Beer Markets: Exploratory Study Examining Public Health Implications of Official Data and Market Intelligence Reports. J. Stud. Alcohol Drugs.

[44] Franco L., Galán C., Bravo R., Bejarano I., Peñas-Lledo E., Rodríguez A.B., Barriga C., Cubero J. (2015). Effect of non-alcohol beer on anxiety: Relationship of 5-HIAA. Neurochem. J..

[45] Franco L., Bravo R., Galan C., Rodríguez A., Barriga C., Cubero J. (2014). Effect of non-alcoholic beer on Subjective Sleep Quality in a university stressed population. Acta Physiol. Hung..

[46] Franco L., Sanchez C.L., Bravo R., Rodríguez A.B., Barriga C., Romero E., Cubero J. (2012). The Sedative Effect of Non-Alcoholic Beer in Healthy Female Nurses. PLoS ONE.

[47] Mateo-Gallego R., Pérez-Calahorra S., Lamiquiz-Moneo I., Marco-Benedí V., Bea A.M., Fumanal A.J., Prieto-Martín A., Laclaustra M., Cenarro A., Civeira F. (2020). Effect of an alcohol-free beer enriched with isomaltulose and a resistant dextrin on insulin resistance in diabetic patients with overweight or obesity. Clin. Nutr..

[48] Macías-Rodríguez R.U., Ruiz-Margáin A., Román-Calleja B.M., Espin-Nasser E.M., Flores-García N.C., Torre A., Galicia-Hernández G., Rios-Torres S.L., Fernández-Del-Rivero G., Orea-Tejeda A. (2020). Clinical Trials Study: Effect of non-alcoholic beer, diet and exercise on endothelial function, nutrition and quality of life in patients with cirrhosis. World J. Hepatol..

[49] Daimiel L., Micó V., Díez-Ricote L., Ruiz-Valderrey P., Istas G., Rodríguez-Mateos A., Ordovás J.M. (2021). Alcoholic and Non-Alcoholic Beer Modulate Plasma and Macrophage microRNAs Differently in a Pilot Intervention in Humans with Cardiovascular Risk. Nutrients.

[50] Chiva-Blanch G., Condines X., Magraner E., Roth I., Valderas-Martínez P., Arranz S., Casas R., Martínez-Huélamo M., Vallverdú-Queralt A., Rada P.Q. (2014). The non-alcoholic fraction of beer increases stromal cell derived factor 1 and the number of circulating endothelial progenitor cells in high cardiovascular risk subjects: A randomized clinical trial. Atherosclerosis.

[51] Chiva-Blanch G., Magraner E., Condines X., Valderas-Martínez P., Roth I., Arranz S., Casas R., Navarro M., Hervas A., Sisó A. (2015). Effects of alcohol and polyphenols from beer on atherosclerotic biomarkers in high cardiovascular risk men: A randomized feeding trial. Nutr. Metab. Cardiovasc. Dis..

[52] Noguer M., Cerezo A.B., Navarro E.D., Garcia-Parrilla M. (2012). Intake of alcohol-free red wine modulates antioxidant enzyme activities in a human intervention study. Pharmacol. Res..

[53] Codoñer-Franch P., Hernandez-Aguilar M.-T., Navarro-Ruiz A., López-Jaén A.B., Borja-Herrero C., Valls-Bellés V. (2013). Diet Supplementation During Early Lactation with Non-alcoholic Beer Increases the Antioxidant Properties of Breastmilk and Decreases the Oxidative Damage in Breastfeeding Mothers. Breastfeed. Med..

[54] Schneider C., Thierauf A., Kempf J., Auwärter V. (2013). Ethanol Concentration in Breastmilk After the Consumption of Non-alcoholic Beer. Breastfeed. Med..

[55] Yin J., Winzenberg T., Quinn S., Giles G., Jones G. (2011). Beverage-specific alcohol intake and bone loss in older men and women: A longitudinal study. Eur. J. Clin. Nutr..

[56] Scherr J., Nieman D.C., Schuster T., Habermann J., Rank M., Braun S., Pressler A., Wolfarth B., Halle M. (2012). Nonalcoholic Beer Reduces Inflammation and Incidence of Respiratory Tract Illness. Med. Sci. Sports Exerc..

[57] Castro-Sepulveda M., Johannsen N., Astudillo S., Jorquera C., Álvarez C., Zbinden-Foncea H., Ramírez-Campillo R. (2016). Effects of Beer, Non-Alcoholic Beer and Water Consumption before Exercise on Fluid and Electrolyte Homeostasis in Athletes. Nutrients.

[58] Desbrow B., Murray D., Leveritt M. (2013). Beer as a Sports Drink? Manipulating Beer’s Ingredients to Replace Lost Fluid. Int. J. Sport Nutr. Exerc. Metab..

[59] Desbrow B., Cecchin D., Jones A., Grant G., Irwin C., Leveritt M. (2015). Manipulations to the Alcohol and Sodium Content of Beer for Postexercise Rehydration. Int. J. Sport Nutr. Exerc. Metab..

[60] Wijnen A.H.C., Steennis J., Catoire M., Wardenaar F.C., Mensink M. (2016). Post-Exercise Rehydration: Effect of Consumption of Beer with Varying Alcohol Content on Fluid Balance after Mild Dehydration. Front. Nutr..

[61] Krennhuber K., Kahr H., Jäger A. (2016). Suitability of Beer as An Alternative to Classical Fitness Drinks. Curr. Res. Nutr. Food Sci. J..

[62] Blackmore H., Hidrio C., Godineau P., Yeomans M.R. (2019). The effect of implicit and explicit extrinsic cues on hedonic and sensory expectations in the context of beer. Food Qual. Prefer..

[63] Fukuda M. (2019). The effects of non-alcoholic beer on response inhibition: An open-label study. Learn. Motiv..

[64] Bucher T., Frey E., Wilczynska M., Deroover K., Dohle S. (2020). Consumer perception and behaviour related to low-alcohol wine: Do people overcompensate?. Public Health Nutr..

[65] Smeets P.A.M., De Graaf C. (2018). Brain Responses to Anticipation and Consumption of Beer with and without Alcohol. Chem. Senses.

[66] Frost R., Quiñones I., Veldhuizen M., Alava J.-I., Small D., Carreiras M. (2015). What Can the Brain Teach Us about Winemaking? An fMRI Study of Alcohol Level Preferences. PLoS ONE.

[67] Naspetti S., Alberti F., Mozzon M., Zingaretti S., Zanoli R. (2020). Effect of information on consumer preferences and willingness-to-pay for sparkling mock wines. Br. Food J..

[68] Lachenmeier D.W., Pflaum T., Nieborowsky A., Mayer S., Rehm J. (2016). Alcohol-free spirits as novel alcohol placebo—A viable approach to reduce alcohol-related harms?. Int. J. Drug Policy.

[69] Saliba A.J., Ovington L.A., Moran C.C. (2013). Consumer demand for low-alcohol wine in an Australian sample. Int. J. Wine Res..

[70] Stanco M., Lerro M., Marotta G. (2020). Consumers’ Preferences for Wine Attributes: A Best-Worst Scaling Analysis. Sustainability.

[71] Paixão J.A., Filho E.T., Bolini H.M.A. (2020). Investigation of Alcohol Factor Influence in Quantitative Descriptive Analysis and in the Time-Intensity Profile of Alcoholic and Non-Alcoholic Commercial Pilsen Beers Samples. Beverages.

[72] Chrysochou P. (2014). Drink to get drunk or stay healthy? Exploring consumers’ perceptions, motives and preferences for light beer. Food Qual. Prefer..

[73] Vasiljevic M., Couturier D.-L., Marteau T.M. (2019). What are the perceived target groups and occasions for wines and beers labelled with verbal and numerical descriptors of lower alcohol strength? An experimental study. BMJ Open.

[74] Silva A.P., Jager G., van Bommel R., van Zyl H., Voss H.-P., Hogg T., Pintado M.M., de Graaf C. (2016). Functional or emotional? How Dutch and Portuguese conceptualise beer, wine and non-alcoholic beer consumption. Food Qual. Prefer..

[75] Silva A.P., Jager G., Voss H.-P., van Zyl H., Hogg T., Pintado M., de Graaf C. (2016). What’s in a name? The effect of congruent and incongruent product names on liking and emotions when consuming beer or non-alcoholic beer in a bar. Food Qual. Prefer..

[76] Vasiljevic M., Coulter L., Petticrew M., Marteau T.M. (2018). Marketing messages accompanying online selling of low/er and regular strength wine and beer products in the UK: A content analysis. BMC Public Health.

[77] Vasiljevic M., Couturier D.L., Marteau T.M. (2018). Impact of low alcohol verbal descriptors on perceived strength: An experimental study. Br. J. Health Psychol..

[78] Vasiljevic M., Couturier D.-L., Frings D., Moss A., Albery I., Marteau T.M. (2018). Impact of lower strength alcohol labeling on consumption: A randomized controlled trial. Health Psychol..

[79] Vasiljevic M., Couturier D., Marteau T.M. (2018). Supplemental Material for Impact on Product Appeal of Labeling Wine and Beer With (a) Lower Strength Alcohol Verbal Descriptors and (b) Percent Alcohol by Volume (%ABV): An Experimental Study. Psychol. Addict. Behav..

[80] Llopis E.J., O’Donnell A., Anderson P. (2021). Impact of price promotion, price, and minimum unit price on household purchases of low and no alcohol beers and ciders: Descriptive analyses and interrupted time series analysis of purchase data from 70, 303 British households, 2015–2018 and first half of 2020. Soc. Sci. Med..

[81] Srivastava P., McLaren K.R., Wohlgenant M., Zhao X. (2014). Disaggregated econometric estimation of consumer demand response by alcoholic beverage types. Aust. J. Agric. Resour. Econ..

[82] Vandenberg B., Jiang H., Livingston M. (2019). Effects of changes to the taxation of beer on alcohol consumption and government revenue in Australia. Int. J. Drug Policy.

[83] European Commission PEFCR for Beer. https://ec.europa.eu/environment/eussd/smgp/pdf/Beer%20PEFCR%20June%202018%20final.pdf.

