# Supplementary material for: Production, Consumption, and Potential Public Health Impact of Low- and No-Alcohol Products: Results of a Scoping Review
Source: Nutrients. 2021 Sep 10;13(9):3153. doi: 10.3390/nu13093153 (PMC8466998; doi:10.3390/nu13093153)
Supplement: Supplementary file 1 [file nutrients-13-03153-s001.zip › nutrients-1337871-supplementary.pdf]

Supplementary Materials

Table S1: Blocks used for searches.

Block 1	Block 2	Block 3	Block 4	Block 5	Block 6	Block 7	Block 8	Block 9
1. "Low alcohol"<br>2. "No alcohol"<br>3. "Zero alcohol"<br>4. "Alcohol-free"<br>5. "Alcohol free"<br>6. Reformulation<br>7. Reduc* ethanol content<br>8. Reduc* ethanol strength<br>9. Reduc* "alcohol strength"<br>10. Reduc* "alcohol content"<br>11. Low strength alcohol<br>12. "non-alcoholic"<br>13. 1 or 2 or 3 or 4 or 5 or 6 or 7 or 8 or 9 or 10 or 11 or 12<br>14. Beer<br>15. Cider<br>16. Wine<br>17. Spirits<br>18. Ready to drink<br>19. Fortified wine<br>20. Fermented beverages<br>21. Intermediate products<br>22. 14 or 15 16 or 17 or 18 or 19 or 20 or 21<br>23. 13 and 22	24. Production<br>25. Development<br>26. Process*<br>27. De-alcohol*<br>28. 24 or 25 or 26 or 27<br>29. 23 and 28	30. Cost<br>31. Benefit<br>32. Cost-benefit<br>33. Cost benefit<br>34. Economic evaluation<br>35. 30 or 31 or 32 or 33 or 34<br>36. 29 and 35	37. Water<br>38. Carbon dioxide<br>39. Greenhouse gas<br>40. Climate change<br>41. Sustainab*<br>42. 37 or 38 or 39 or 40 or 41<br>43. 29 and 42	44. Consumption<br>45. Purchase<br>46. Health<br>47. Health benefit<br>48. Health harm<br>49. Risk<br>50. Cancer<br>51. Injury<br>52. Accident<br>53. Alcohol-related harm<br>54. Alcohol related harm<br>55. 44 or 45 or 46 or 47 or 48 or 49 or 50 or 51 or 52 or 53 or 54<br>56. 23 and 55	57. Initiation<br>58. Youth<br>59. Underage drinking<br>60. Brand loyalty<br>61. Brand*<br>62. Inequality<br>63. Drink more<br>64. Consumer surveys<br>65. Accept*<br>66. 57 or 58 or 59 or 60 or 61 or 62 or 63 or 64 or 65<br>67. 23 and 66<br>68. 23 and 83	69. Price<br>70. Tax<br>71. Availability<br>72. Placement<br>73. Minimum unit price<br>74. MUP<br>75. Advertising<br>76. Marketing<br>77. Commercial communication<br>78. Excise duty<br>79. European Commission<br>80. Affordable<br>81. Promotion<br>82. Restrictions<br>83. Circumvent<br>84. 68 or 69 or 70 or 71 or 72 or 73 or 74 or 75 or 76 or 77 or 78 or 79 or 80 or 81 or 82	85. Producer<br>86. Company<br>87. Industry<br>88. Goal<br>89. Target<br>90. Commit*<br>91. Supermarket<br>92. Retail<br>93. 85 or 86 or 87 or 88 or 89 or 90 or 91 or 92<br>94. 23 and 93	95. life cycle assessment<br>96. life cycle"<br>97. "life cycle analysis"<br>98. LCA<br>99. 95 or 96 or 97 or 98<br>100. 23 and 99

Table S2: Search strategy and hits

	Blocks	Search strings (Example PubMed)	PubMed	Web of Science	After removal of duplicates	Selected for full text inspection	Final selection
Search 1	Block 1 + Block 2	(“Low alcohol” OR “No alcohol” OR “Zero alcohol” OR “Alcohol-free” OR “Alcohol free” OR Reformulation OR Reduc* ethanol content OR Reduc* ethanol strength OR Reduc* "alcohol strength" OR Reduc* “alcohol content” OR Low strength alcohol OR “non-alcoholic”) AND (beer OR cider OR wine OR spirits OR ready to drink OR fortified wine OR fermented beverages OR intermediate products) AND (Production OR Development OR Process* OR De-alcohol*) AND (English[Language]) AND (("2011/01/01"[Date - Publication] : "3000"[Date - Publication]))	241	655	746	45	36
Search 2	Block 1 + Block 2 + Block 3	(“Low alcohol” OR “No alcohol” OR “Zero alcohol” OR “Alcohol-free” OR “Alcohol free” OR Reformulation OR Reduc* ethanol content OR Reduc* ethanol strength OR Reduc* "alcohol strength" OR Reduc* “alcohol content” OR Low strength alcohol OR “non-alcoholic”) AND (beer OR cider OR wine OR spirits OR ready to drink OR fortified wine OR fermented beverages OR intermediate products) AND (Production OR Development OR Process* OR De-alcohol*) AND (beer OR cider OR wine OR spirits OR ready to drink OR fortified wine OR fermented beverages OR intermediate products) AND (Production OR Development OR Process* OR De-alcohol*) AND (Cost OR Benefit OR Cost-benefit OR Cost benefit OR economic evaluation) AND (English[Language]) AND (("2011/01/01"[Date - Publication] : "3000"[Date - Publication]))	48	92	127	10	9
Search 3	Block 1 + Block 2 + Block 4	(“Low alcohol” OR “No alcohol” OR “Zero alcohol” OR “Alcohol-free” OR “Alcohol free” OR Reformulation OR Reduc* ethanol content OR Reduc* ethanol strength OR Reduc* "alcohol strength" OR Reduc* “alcohol content” OR Low strength alcohol OR “non-alcoholic”) AND (beer OR cider OR wine OR spirits OR ready to drink OR fortified wine OR fermented beverages OR intermediate products) AND (beer OR cider OR wine OR spirits OR ready to drink OR fortified wine OR fermented beverages OR intermediate products) AND (Production OR Development OR Process* OR De-alcohol*) AND (Water OR Carbon dioxide OR Greenhouse gas OR Climate change OR Sustainab*) AND (English[Language]) AND (("2011/01/01"[Date - Publication] : "3000"[Date - Publication]))	38	150	172	13	11
Search 4	Block 1 + Block 5	(“Low alcohol” OR “No alcohol” OR “Zero alcohol” OR “Alcohol-free” OR “Alcohol free” OR Reformulation OR Reduc* ethanol content OR Reduc* ethanol strength OR Reduc* "alcohol strength" OR Reduc* “alcohol content” OR Low strength alcohol OR ““non-alcoholic””) AND (beer OR cider OR wine OR spirits OR ready to drink OR fortified wine OR fermented beverages OR intermediate products) AND (Consumption OR Purchase OR Health OR Health benefit OR Health harm OR Risk OR Cancer OR Injury OR Accident OR Alcohol-related harm OR Alcohol related harm) AND (English[Language]) AND (("2011/01/01"[Date - Publication] : "3000"[Date - Publication]))	228	494	570	61	48
Search 5	Block 1 + Block 6	(“Low alcohol” OR “No alcohol” OR “Zero alcohol” OR “Alcohol-free” OR “Alcohol free” OR Reformulation OR Reduc* ethanol content OR Reduc* ethanol strength OR Reduc* "alcohol strength" OR Reduc* “alcohol content” OR Low strength alcohol OR “non-alcoholic”) AND (beer OR cider OR wine OR spirits OR ready to drink OR fortified wine OR fermented beverages OR intermediate products) AND (Initiation OR Youth OR Underage drinking OR Brand loyalty OR Brand* OR Inequality OR Drink more OR Consumer survey OR Accept*) AND ((English[Language]) AND ("2011/01/01"[Date - Publication] : "3000"[Date - Publication]))	105	261	309	36	27

Search 6	Block 1 + Block 7	(“Low alcohol” OR “No alcohol” OR “Zero alcohol” OR “Alcohol-free” OR “Alcohol free” OR Reformulation OR Reduc* ethanol content OR Reduc* ethanol strength OR Reduc* "alcohol strength" OR Reduc* “alcohol content” OR Low strength alcohol OR “non-alcoholic”) AND (beer OR cider OR wine OR spirits OR ready to drink OR fortified wine OR fermented beverages OR intermediate products) AND (Price OR Tax OR Availability OR Placement OR Minimum unit price OR MUP OR Advertising OR Marketing OR Commercial communication OR Excise duty OR European Commission OR Affordable OR Promotion OR Restrictions OR Circumvent) AND (English[Language]) AND (("2011/01/01"[Date - Publication] : "3000"[Date - Publication]))	100	146	204	29	25
Search 7	Block 1 + Block 8	(“Low alcohol” OR “No alcohol” OR “Zero alcohol” OR “Alcohol-free” OR “Alcohol free” OR Reformulation OR Reduc* ethanol content OR Reduc* ethanol strength OR Reduc* "alcohol strength" OR Reduc* “alcohol content” OR Low strength alcohol OR “non-alcoholic”) AND (beer OR cider OR wine OR spirits OR ready to drink OR fortified wine OR fermented beverages OR intermediate products) AND (Producer OR Company OR Industry OR Goal OR Target OR Commit* OR Supermarket OR Retail) AND (English[Language]) AND (("2011/01/01"[Date - Publication] : "3000"[Date - Publication]))	163	303	396	29	23
Search 8	Block 1 + 9	(“Low alcohol” OR “No alcohol” OR “Zero alcohol” OR “Alcohol-free” OR “Alcohol free” OR Reformulation OR Reduc* ethanol content OR Reduc* ethanol strength OR Reduc* "alcohol strength" OR Reduc* “alcohol content” OR Low strength alcohol OR “non-alcoholic”) AND (beer OR cider OR wine OR spirits OR ready to drink OR fortified wine OR fermented beverages OR intermediate products) AND (“life cycle assessment” OR “life cycle” OR “life cycle analysis” OR LCA) AND (English[Language]) AND (("2011/01/01"[Date - Publication] : "3000"[Date - Publication]))<br>ALL=(“Low alcohol” OR “No alcohol” OR “Zero alcohol” OR “Alcohol-free” OR “Alcohol free” OR Reformulation OR Reduc* ethanol content OR Reduc* ethanol strength OR Reduc* "alcohol strength" OR Reduc* “alcohol content” OR Low strength alcohol OR “non-alcoholic”) AND ALL=(beer OR cider OR wine OR spirits OR ready to drink OR fortified wine OR fermented beverages OR intermediate products) AND ALL=(“life cycle assessment” OR “life cycle” OR “life cycle analysis” OR LCA)	2	6	7	2	1
Total search hits			923	2101	1116*	82*	65
Additional papers found**							5

*(after removal also of duplicates between searches)

** from other sources (through Google Scholar, existing library)

Table S3: Papers' appearances in searches

	**Search group**
Anderson, P., Jané Llopis, E., O'Donnell, A., Manthey, J., & Rehm, J. (2020). Impact of low and no alcohol beers on purchases of alcohol: interrupted time series analysis of British household shopping data, 2015-2018. Bmj Open, 10(10), e036371. doi:10.1136/bmjopen-2019-036371	S1 S4
Anderson, P., Llopis, E. J., & Rehm, J. (2020). Evaluation of Alcohol Industry Action to Reduce the Harmful Use of Alcohol: Case Study from Great Britain. Alcohol and Alcoholism, 55(4), 424-432. doi:10.1093/alcalc/agaa029	S4 S6 S7
Baiano, A. (2020). Craft beer: An overview. Compr Rev Food Sci Food Saf. doi:10.1111/1541-4337.12693	S1 S3 S4 S6 S7
Baschali, A., Tsakalidou, E., Kyriacou, A., Karavasiloglou, N., & Matalas, A. L. (2017). Traditional low-alcoholic and non-alcoholic fermented beverages consumed in European countries: a neglected food group. Nutrition Research Reviews, 30(1), 1-24. doi:10.1017/s0954422416000202	S3
Blackmore, H., Hidrio, C., Godineau, P., & Yeomans, M. R. (2020). The effect of implicit and explicit extrinsic cues on hedonic and sensory expectations in the context of beer. Food Quality and Preference, 81. doi:10.1016/j.foodqual.2019.103855	S4
Blanco, C. A., Andrés-Iglesias, C., & Montero, O. (2016). Low-alcohol Beers: Flavor Compounds, Defects, and Improvement Strategies. Crit Rev Food Sci Nutr, 56(8), 1379-1388. doi:10.1080/10408398.2012.733979	S1 S6 S7
Branyik, T., Silva, D. P., Baszczynski, M., Lehnert, R., & Silva, J. (2012). A review of methods of low alcohol and alcohol-free beer production. Journal of Food Engineering, 108(4), 493-506. doi:10.1016/j.jfoodeng.2011.09.020	S1 S4 S5 S7
Bucher, T., Deroover, K., & Stockley, C. (2018). Low-Alcohol Wine: A Narrative Review on Consumer Perception and Behaviour. Beverages, 4(4). doi:10.3390/beverages4040082	S4 S5 S6
Bucher, T., Frey, E., Wilczynska, M., Deroover, K., & Dohle, S. (2020). Consumer perception and behaviour related to low-alcohol wine: do people overcompensate? Public Health Nutrition, 23(11), 1939-1947. doi:10.1017/s1368980019005238	S6
Castro-Sepulveda, M., Johannsen, N., Astudillo, S., Jorquera, C., Alvarez, C., Zbinden-Foncea, H., & Ramirez-Campillo, R. (2016). Effects of Beer, Non-Alcoholic Beer and Water Consumption before Exercise on Fluid and Electrolyte Homeostasis in Athletes. Nutrients, 8(6). doi:10.3390/nu8060345	S4 S5
Catarino, M., & Mendes, A. (2011). Non-alcoholic beer-A new industrial process. Separation and Purification Technology, 79(3), 342-351. doi:10.1016/j.seppur.2011.03.020	S3 S7
Chiva-Blanch, G., Condines, X., Magraner, E., Roth, I., Valderas-Martínez, P., Arranz, S., . . . Estruch, R. (2014). The non-alcoholic fraction of beer increases stromal cell derived factor 1 and the number of circulating endothelial progenitor cells in high cardiovascular risk subjects: a randomized clinical trial. Atherosclerosis, 233(2), 518-524. doi:10.1016/j.atherosclerosis.2013.12.048	S4
Chiva-Blanch, G., Magraner, E., Condines, X., Valderas-Martínez, P., Roth, I., Arranz, S., . . . Estruch, R. (2015). Effects of alcohol and polyphenols from beer on atherosclerotic biomarkers in high cardiovascular risk men: a randomized feeding trial. Nutr Metab Cardiovasc Dis, 25(1), 36-45. doi:10.1016/j.numecd.2014.07.008	S4
Chrysochou, P. (2014). Drink to get drunk or stay healthy? Exploring consumers' perceptions, motives and preferences for light beer. Food Quality and Preference, 31, 156-163. doi:10.1016/j.foodqual.2013.08.006	S4 S5
Codoner-Franch, P., Hernandez-Aguilar, M. T., Navarro-Ruiz, A., Lopez-Jaen, A. B., Borja-Herrero, C., & Valls-Belles, V. (2013). Diet Supplementation During Early Lactation with Non-alcoholic Beer Increases the Antioxidant Properties of Breastmilk and Decreases the Oxidative Damage in Breastfeeding Mothers. Breastfeeding Medicine, 8(2), 164-169. doi:10.1089/bfm.2012.0059	S1 S4
Daimiel, L., Mico, V., Diez-Ricote, L., Ruiz-Valderrey, P., Istas, G., Rodriguez-Mateos, A., & Ordovas, J. M. (2021). Alcoholic and Non-Alcoholic Beer Modulate Plasma and Macrophage microRNAs Differently in a Pilot Intervention in Humans with Cardiovascular Risk. Nutrients, 13(1). doi:10.3390/nu13010069	S1 S2 S4
Dequin, S., Escudier, J. L., Bely, M., Noble, J., Albertin, W., Masneuf-Pomarede, I., . . . Sablayrolles, J. M. (2017). How to adapt winemaking practices to modified grape composition under climate change conditions. Oeno One, 51(2), 205-214. doi:10.20870/oeno-one.2016.0.0.1584	S1 S3
Desbrow, B., Cecchin, D., Jones, A., Grant, G., Irwin, C., & Leveritt, M. (2015). Manipulations to the Alcohol and Sodium Content of Beer for Postexercise Rehydration. International Journal of Sport Nutrition and Exercise Metabolism, 25(3), 262-270. doi:10.1123/ijsnem.2014-0064	S4 S5

Desbrow, B., Murray, D., & Leveritt, M. (2013). Beer as a Sports Drink? Manipulating Beer's Ingredients to Replace Lost Fluid. International Journal of Sport Nutrition and Exercise Metabolism, 23(6), 593-600. doi:10.1123/ijsnem.23.6.593	S4 S5
Franco, L., Bravo, R., Galan, C., Rodriguez, A. B., Barriga, C., & Cubero, J. (2014). Effect of non-alcoholic beer on Subjective Sleep Quality in a university stressed population. Acta Physiologica Hungarica, 101(3), 353-361. doi:10.1556/APhysiol.101.2014.3.10	S4
Franco, L., Galan, C., Bravo, R., Bejarano, I., Penas-Lledo, E., Rodriguez, A. B., . . . Cubero, J. (2015). Effect of non-alcohol beer on anxiety: Relationship of 5-HIAA. Neurochemical Journal, 9(2), 149-152. doi:10.1134/s181971241502004x	S4 S7
Franco, L., Sánchez, C., Bravo, R., Rodríguez, A. B., Barriga, C., Romero, E., & Cubero, J. (2012). The sedative effect of non-alcoholic beer in healthy female nurses. Plos One, 7(7), e37290. doi:10.1371/journal.pone.0037290	S1 S2 S4
Frost, R., Quinones, I., Veldhuizen, M., Alava, J. I., Small, D., & Carreiras, M. (2015). What Can the Brain Teach Us about Winemaking? An fMRI Study of Alcohol Level Preferences. Plos One, 10(3). doi:10.1371/journal.pone.0119220	S1
Fukuda, M. (2019). The effects of non-alcoholic beer on response inhibition: An open-label study. Learning and Motivation, 66, 46-54. doi:10.1016/j.lmot.2019.04.002	S1 S3 S5
Hagemann, M. H., Bogner, K., Marchioni, E., & Braun, S. (2016). Chances for dry-hopped non-alcoholic beverages? Part 1: Concept and market prospects. Brewing Science, 69(7-8), 50-55. Retrieved from <Go to ISI>://WOS:000413980700002	S1 S4 S6
Hagemann, M. H., Schmidt-Cotta, V., Marchioni, E., & Braun, S. (2017). Chance for Dry-hopped Non-alcoholic Beverages? Part 2: Health Properties and Target Consumers. Brewing Science, 70(7-8), 118-123. doi:10.23763/BrSc17-10Hagemann	S4 S6 S7
Jackowski, M., & Trusek, A. (2018). Non-alcoholic beer production - an overview. Polish Journal of Chemical Technology, 20(4), 32-38. doi:10.2478/pjct-2018-0051	S1
Krennhuber, K., Kahr, H., & Jager, A. (2016). Suitability of Beer as an Alternative to Classical Fitness Drinks. Current Research in Nutrition and Food Science, 4, 26-31. doi:10.12944/CRNFSJ.4.Special-Issue-October.04	S5
Kypri, K., Harrison, S., & McCambridge, J. (2020). Ethanol Content in Australian and New Zealand Beer Markets: Exploratory Study Examining Public Health Implications of Official Data and Market Intelligence Reports. Journal of Studies on Alcohol and Drugs, 81(3), 320-330. Retrieved from <Go to ISI>://WOS:000600455400004	S1 S4 S6 S7
Lachenmeier, D. W., Pflaum, T., Nieborowsky, A., Mayer, S., & Rehm, J. (2016). Alcohol-free spirits as novel alcohol placebo - A viable approach to reduce alcohol-related harms? International Journal of Drug Policy, 32, 1-2. doi:10.1016/j.drugpo.2016.04.001	S4
Laoretani, D. S., Sanchez, R. J., Paredes, D. A. F., Iribarren, O. A., & Espinosa, J. (2020). On the conceptual modeling, economic analysis and life cycle assessment of partial dealcoholization alternatives of bitter extracts. Separation and Purification Technology, 251. doi:10.1016/j.seppur.2020.117331	S1 S2 S3 S4 S6
Liguori, L., Albanese, D., Crescitelli, A., Di Matteo, M., & Russo, P. (2019). Impact of dealcoholization on quality properties in white wine at various alcohol content levels. Journal of Food Science and Technology-Mysore, 56(8), 3707-3720. doi:10.1007/s13197-019-03839-x	S7
Llopis, E. J., O'Donnell, A., & Anderson, P. (2021). Impact of price promotion, price, and minimum unit price on household purchases of low and no alcohol beers and ciders: Descriptive analyses and interrupted time series analysis of purchase data from 70, 303 British households, 2015-2018 and first half of 2020. Soc Sci Med, 270, 113690. doi:10.1016/j.socscimed.2021.113690	S1 S2 S4 S6 S7
Longo, R., Blackman, J. W., Antalick, G., Torley, P. J., Rogiers, S. Y., & Schmidtke, L. M. (2018). A comparative study of partial dealcoholisation versus early harvest: Effects on wine volatile and sensory profiles. Food Chemistry, 261, 21-29. doi:10.1016/j.foodchem.2018.04.013	S1 S7
Longo, R., Blackman, J. W., Antalick, G., Torley, P. J., Rogiers, S. Y., & Schmidtke, L. M. (2018). Harvesting and blending options for lower alcohol wines: a sensory and chemical investigation. Journal of the Science of Food and Agriculture, 98(1), 33-42. doi:10.1002/jsfa.8434	S1 S7
Longo, R., Blackman, J. W., Torley, P. J., Rogiers, S. Y., & Schmidtke, L. M. (2017). Changes in volatile composition and sensory attributes of wines during alcohol content reduction. J Sci Food Agric, 97(1), 8-16. doi:10.1002/jsfa.7757	S1 S4 S5 S7
Macias-Rodriguez, R. U., Ruiz-Margain, A., Roman-Calleja, B. M., Espin-Nasser, M. E., Flores-Garcia, N. C., Torre, A., . . . Lozano-Cruz, O. A. (2020). Effect of non-alcoholic beer, diet and exercise on endothelial function, nutrition and quality of life in patients with cirrhosis. World Journal of Hepatology, 12(12), 1299-1313. doi:10.4254/wjh.v12.i12.1299	S1 S2 S3 S4 S7

Mangindaan, D., Khoiruddin, K., & Wenten, I. G. (2018). Beverage dealcoholization processes: Past, present, and future. Trends in Food Science & Technology, 71, 36-45. doi:10.1016/j.tifs.2017.10.018	S1 S4 S5 S6
Margallo, M., Aldaco, R., Barcelo, A., Diban, N., Ortiz, I., & Irabien, A. (2015). Life cycle assessment of technologies for partial dealcoholisation of wines. Sustainable Production and Consumption, 2, 29-39. doi:10.1016/j.spc.2015.07.007	S1 S2 S3 S4 S5
Mateo-Gallego, R., Perez-Calahorra, S., Lamiquiz-Moneo, I., Marco-Benedi, V., Bea, A. M., Fumanal, A. J., . . . Civeira, F. (2020). Effect of an alcohol-free beer enriched with isomaltulose and a resistant dextrin on insulin resistance in diabetic patients with overweight or obesity. Clinical Nutrition, 39(2), 475-483. doi:10.1016/j.clnu.2019.02.025	S1 S4
Mellor, D. D., Hanna-Khalil, B., & Carson, R. (2020). A review of the potential health benefits of low alcohol and alcohol-free beer: Effects of ingredients and craft brewing processes on potentially bioactive metabolites. *Beverages*, *6*(2), 25.	A
Muller, C., Neves, L. E., Gomes, L., Guimaraes, M., & Ghesti, G. (2020). Processes for alcohol-free beer production: a review. Food Science and Technology, 40(2), 273-281. doi:10.1590/fst.32318	S1 S6
Myles, C. C., Goff, P. D., Wiley, D., & Savelyev, A. (2020). Low Gravity on the Rise: A Sociocultural Examination of Low Alcohol Beer in the United States. In *The Geography of Beer* (pp. 87-100). Springer, Cham.	A
Naspetti, S., Alberti, F., Mozzon, M., Zingaretti, S., & Zanoli, R. (2020). Effect of information on consumer preferences and willingness-to-pay for sparkling mock wines. British Food Journal, 122(8), 2621-2638. doi:10.1108/bfj-06-2019-0469	S1 S4 S5 S6
Noguer, M. A., Cerezo, A. B., Donoso Navarro, E., & Garcia-Parrilla, M. C. (2012). Intake of alcohol-free red wine modulates antioxidant enzyme activities in a human intervention study. Pharmacol Res, 65(6), 609-614. doi:10.1016/j.phrs.2012.03.003	S4
Ozturk, B., & Anli, E. (2014). Different techniques for reducing alcohol levels in wine: A review. In J. M. Aurand (Ed.), 37th World Congress of Vine and Wine and 12th General Assembly of the Oiv (Vol. 3).	S1 S2 S5 S6
Paixão, J. A., Tavares Filho, E., & Bolini, H. M. A. (2020). Investigation of Alcohol Factor Influence in Quantitative Descriptive Analysis and in the Time-Intensity Profile of Alcoholic and Non-Alcoholic Commercial Pilsen Beers Samples. *Beverages*, *6*(4), 73.	A
Paredes, D. A. F., Laoretani, D. S., Morero, B., Sánchez, R. J., Iribarren, O. A., & Espinosa, J. (2020). Screening of membrane technologies in concentration of bitter extracts with simultaneous alcohol recovery: An approach including both economic and environmental issues. *Separation and Purification Technology*, *237*, 116339.	S8
Rehm, J., Lachenmeier, D. W., Llopis, E. J., Imtiaz, S., & Anderson, P. (2016). Evidence of reducing ethanol content in beverages to reduce harmful use of alcohol. Lancet Gastroenterology & Hepatology, 1(1), 78-83. doi:10.1016/s2468-1253(16)30013-9	S4 S5 S7
Salanta, L. C., Coldea, T. E., Ignat, M. V., Pop, C. R., Tofana, M., Mudura, E., . . . Zhao, H. F. (2020). Non-Alcoholic and Craft Beer Production and Challenges. Processes, 8(11). doi:10.3390/pr8111382	S1 S2 S3 S5 S6
Saliba, A. J., Ovington, L. A., & Moran, C. C. (2013). Consumer demand for low-alcohol wine in an Australian sample. *International Journal of Wine Research*, *5*, 1-8.	A
Scherr, J., Nieman, D. C., Schuster, T., Habermann, J., Rank, M., Braun, S., ... & Halle, M. (2012). Nonalcoholic beer reduces inflammation and incidence of respiratory tract illness. *Medicine & Science in Sports & Exercise*, *44*(1), 18-26.	A
Schneider, C., Thierauf, A., Kempf, J., & Auwarter, V. (2013). Ethanol Concentration in Breastmilk After the Consumption of Non-alcoholic Beer. Breastfeeding Medicine, 8(3), 291-293. doi:10.1089/bfm.2012.0156	S4 S5
Silva, A. P., Jager, G., van Bommel, R., van Zyl, H., Voss, H. P., Hogg, T., . . . de Graaf, C. (2016). Functional or emotional? How Dutch and Portuguese conceptualise beer, wine and non-alcoholic beer consumption. Food Quality and Preference, 49, 54-65. doi:10.1016/j.foodqual.2015.11.007	S4 S5
Silva, A. P., Jager, G., Van Zyl, H., Voss, H. P., Pintado, M., Hogg, T., & De Graaf, C. (2017). Cheers, proost, saúde: Cultural, contextual and psychological factors of wine and beer consumption in Portugal and in the Netherlands. Crit Rev Food Sci Nutr, 57(7), 1340-1349. doi:10.1080/10408398.2014.969396	S4 S6 S7
Silva, A. P., Jager, G., Voss, H. P., van Zyl, H., Hogg, T., Pintado, M., & de Graaf, C. (2017). What's in a name? The effect of congruent and incongruent product names on liking and emotions when consuming beer or non-alcoholic beer in a bar (vol 55, pg 58, 2017). Food Quality and Preference, 61, 78-78. doi:10.1016/j.foodqual.2017.03.003	S4 S5

Smeets, P. A. M., & de Graaf, C. (2019). Brain Responses to Anticipation and Consumption of Beer with and without Alcohol. Chemical Senses, 44(1), 51-60. doi:10.1093/chemse/bjy071	S1 S3 S4 S6
Srivastava, P., McLaren, K. R., Wohlgenant, M., & Zhao, X. Y. (2015). Disaggregated econometric estimation of consumer demand response by alcoholic beverage types. Australian Journal of Agricultural and Resource Economics, 59(3), 412-432. doi:10.1111/1467-8489.12095	S4 S5 S6
Stanco, M., Lerro, M., & Marotta, G. (2020). Consumers' Preferences for Wine Attributes: A Best-Worst Scaling Analysis. Sustainability, 12(7). doi:10.3390/su12072819	S4 S5 S6
Vandenberg, B., Jiang, H., & Livingston, M. (2019). Effects of changes to the taxation of beer on alcohol consumption and government revenue in Australia. International Journal of Drug Policy, 70, 1-7. doi:10.1016/j.drugpo.2019.04.012	S1 S2 S4 S6
Varela, C., Dry, P. R., Kutyna, D. R., Francis, I. L., Henschke, P. A., Curtin, C. D., & Chambers, P. J. (2015). Strategies for reducing alcohol concentration in wine. Australian Journal of Grape and Wine Research, 21, 670-679. doi:10.1111/ajgw.12187	S1 S4 S6 S7
Varela, J., & Varela, C. (2019). Microbiological strategies to produce beer and wine with reduced ethanol concentration. Curr Opin Biotechnol, 56, 88-96. doi:10.1016/j.copbio.2018.10.003	S6 S7
Vasiljevic, M., Coulter, L., Petticrew, M., & Marteau, T. M. (2018). Marketing messages accompanying online selling of low/er and regular strength wine and beer products in the UK: a content analysis. Bmc Public Health, 18. doi:10.1186/s12889-018-5040-6	S1 S4 S5 S6 S7
Vasiljevic, M., Couturier, D. L., & Marteau, T. M. (2018). Impact of low alcohol verbal descriptors on perceived strength: An experimental study. British journal of health psychology, 23(1), 38-67. doi:10.1111/bjhp.12273	S1 S4 S5 S7
Vasiljevic, M., Couturier, D. L., & Marteau, T. M. (2018). Impact on Product Appeal of Labeling Wine and Beer With (a) Lower Strength Alcohol Verbal Descriptors and (b) Percent Alcohol by Volume (%ABV): An Experimental Study. Psychology of Addictive Behaviors, 32(7), 779-791. doi:10.1037/adb0000376	S1 S4 S5 S7
Vasiljevic, M., Couturier, D. L., & Marteau, T. M. (2019). What are the perceived target groups and occasions for wines and beers labelled with verbal and numerical descriptors of lower alcohol strength? An experimental study. Bmj Open, 9(6). doi:10.1136/bmjopen-2018-024412	S1 S4 S5 S6 S7
Vasiljevic, M., Couturier, D. L., Frings, D., Moss, A. C., Albery, I. P., & Marteau, T. M. (2018). Impact of Lower Strength Alcohol Labeling on Consumption: A Randomized Controlled Trial. Health Psychology, 37(7), 658-667. doi:10.1037/hea0000622	S1 S4 S5 S6 S7
Wijnen, A. H., Steennis, J., Catoire, M., Wardenaar, F. C., & Mensink, M. (2016). Post-Exercise Rehydration: Effect of Consumption of Beer with Varying Alcohol Content on Fluid Balance after Mild Dehydration. Front Nutr, 3, 45. doi:10.3389/fnut.2016.00045	S1 S3 S4 S5
Yin, J., Winzenberg, T., Quinn, S., Giles, G., & Jones, G. (2011). Beverage-specific alcohol intake and bone loss in older men and women: a longitudinal study. European Journal of Clinical Nutrition, 65(4), 526-532. doi:10.1038/ejcn.2011.9	S4
Zendeboodi, F., Jannat, B., Sohrabvandi, S., Khanniri, E., Mortazavian, A. M., Khosravi, K., . . . Esmaeili, S. (2019). Monitoring of ethanol content in non-alcoholic beer stored in different packages under different storage temperatures. Biointerface Research in Applied Chemistry, 9(6), 4624-4628. doi:10.33263/briac96.624628	S1 S4 S5

S1 – Search 1; S2- Search 2; S3 – Search 3; S4 – Search 4; S5 – Search 5; S6 – Search 6; S7 – Search 7; S8 – Search 8; A - Additional

Table S4: Summary of papers across five themes

Topic	Reference	Focus of the paper	Definition of no and low alcohol product	Methods	Key findings
**Production processes**					
**Beer**	Blanco, C. A., Andrés-Iglesias, C., & Montero, O. (2016). Low-alcohol Beers: Flavor Compounds, Defects, and Improvement Strategies. Crit Rev Food Sci Nutr, 56(8), 1379-1388. doi:10.1080/10408398.2012.733979	To show the dealcoholization processes that are commonly used to reduce the alcohol content in beer, their negative consequences to beer flavour, and strategies to circumvent such negative consequences	Regulations in EU countries: alcohol free beers less than or equal to 0.5% ABV and low alcohol beers (LAB) with no more than 1.2% ABV	Review	When ethanol is removed from regular beer, there are four consequences for LABs:<br>• –In incompleted fermentation, carbonyl compounds are reduced only slightly, therefore conferring unpleasant flavors.<br>• A lack of flavor due to the elimination of both ethanol and other alcohols during the dealcoholization process.<br>• –Some favorable compounds are missing because ethanol operates as a solvent.<br>• LAB contamination with spoilage microorganisms increase due to the lack of ethanol<br><br>Currently investigated solutions to negative consequences of dealcholization processes are:<br>• Control strategies based on the manipulation of parameters during fermentation<br>• Use of special yeast strains that form less ethanol during complete fermentation of wort sugars<br>• Emerging technologies to produce non-alcoholic beers by removing ethanol from a completely fermented beer.
	Branyik, T., Silva, D. P., Baszczynski, M., Lehnert, R., & Silva, J. (2012). A review of methods of low alcohol and alcohol-free beer production. Journal of Food Engineering, 108(4), 493-506. doi:10.1016/j.jfoodeng.2011.09.020	To present overview and comparison of two techniques within strategies of producing beers with low alcohol content (gentle removal of alcohol from regular beer and by limited ethanol formation during the beer fermentation), provide an evaluation of sensorial properties of low-alcohol and an alcohol-free beer produced, and suggest possibilities for their additional improvement.	Regulations in EU countries: alcohol free beers less than or equal to 0.5% ABV and low alcohol beers (LAB) with no more than 1.2% ABV	Review	Two main strategies; removal of alcohol from regular beer (physical method) and by limited ethanol formation during the beer fermentation (biological process).<br><br>Production of alcohol-free beer by ethanol removal methods can be through thermal processes or membrane processes, and production of alcohol-free beer by methods of restricted ethanol formation can be achieved through changed mashing process, arrested or limited fermentation process, use of special yeast or continuous fermentation.

					A reliable and comprehensive economic comparison of various methods of the LAB/AFB production is not available and therefore it is impossible to define the best process
	Catarino, M., & Mendes, A. (2011). Non-alcoholic beer-A new industrial process. Separation and Purification Technology, 79(3), 342-351. doi:10.1016/j.seppur.2011.03.020	To evaluate a new industrial process for producing non-alcoholic beer with a corrected natural flavour profile, and investigate the operating conditions of the studied unit.	Non-alcoholic beer (l<0.5vol.%)	Review	High permeation temperature and low feed flowrate are the most effective for maximizing the permeation flux and the equilibrium of the flavour profile. The aroma depleted beer stream is then added to the feed stream of an industrial unit of spinning cone column distillation for dealcoholization. In this unit, the beverage contacts counter-currently with a water vapour stream that strips the ethanol and other volatile aroma compounds from beer. After dealcoholization, the beer is blended with the extracted aroma compounds and with a fraction of original beer to achieve a non-alcoholic beer (ethanol lower than 0.5 vol.%) with a good flavour profile. This new industrial process proved to originate a dealcoholized beer with a taste very close to the original one.
	Jackowski, M., & Trusek, A. (2018). Non-alcoholic beer production - an overview. Polish Journal of Chemical Technology, 20(4), 32-38. doi:10.2478/pjct-2018-0051	To present two major approaches to obtain soft drink with similar organoleptic properties as standard beer	Regulations differ by countries:<br>Poland, Germany, USA, China: non-alcoholic beer should contain no more than 0.5% ABV<br>Spain: less than or equal 1% ABV<br>France: less than or equal 1.2%ABV	Review	First approach is to interfere with biological aspects of beer production technology like changes in mashing regime or to perform fermentation in conditions that pro-mote lower alcohol production or using special (often genetic modifi ed) microorganism.<br><br>Second approach is to remove alcohol from standard beer, It is mainly possible due to evaporation techniques and membrane ones.
	Muller, C., Neves, L. E., Gomes, L., Guimaraes, M., & Ghesti, G. (2020). Processes for alcohol-free beer production: a review. Food Science and Technology, 40(2), 273-281. doi:10.1590/fst.32318	To review processes for alcohol-free beer production	Regulations differ by countries:.<br>Germany, Brazil the term Alkoholfrei, or without alcohol, is applied in the same parameters defined for Brazilian legislation, i.e. beers containing up to 0.5% alcohol by volume.	Review	The aim on alcohol-free beer production is to have products that taste similarly to their alcoholic equivalents, the sole absence of alcohol and low concentrations of esters and higher alcohols and the higher concentration of aldehydes create a “worty” organoleptic impression of those products.

			USA: the term alcohol-free beer is exclusive to products which have no detectable residual alcohol content, whereas products with an alcohol content of up to 0.5% by volume are referred to as non-alcoholic.<br>UK: it may only be termed non-alcoholic beer, if it contains up to 0.05% residual alcohol content, whereas they are referred to as dealcoholized products if its alcoholic strength is between 0.05% and 0.5% by volume and of low-alcohol (low alcoholic strength) with an alcoholic strength of 0.5% to 1.2% by volume.		Alcohol-free beers can be obtained by the restriction of alcohol formation during fermentation, the use of special yeast strains that consume or don't produce alcohol, the thermal removal of alcohol and by the removal of alcohol trough membrane processes.<br>Membrane separated alcohol-free beers preserve more of the natural constituents of beer aroma and those products usually taste better than those obtained through other processes.<br>Pervaporation techniques show a broad spectrum of use and can even be combined with other processes and perform the removal of undesired products or separation and recuperation of aromatic substances
	Salanta, L. C., Coldea, T. E., Ignat, M. V., Pop, C. R., Tofana, M., Mudura, E., . . . Zhao, H. F. (2020). Non-Alcoholic and Craft Beer Production and Challenges. Processes, 8(11). doi:10.3390/pr8111382	To review the production technologies involved in in production of non-alcoholic and craft beer in terms of efficiency and production costs	non-alcoholic-beers (NAB): low-alcohol beers (<2.5%,v/v) and alcohol-free beers (≤1%v/v).<br><br>Low-alcohol beer (LAB):. In Germany, the USA, and in China, this limit is no more than 0.5% v/v; in Spain, the maximum value is 1%v/v alcohol; while in France, is of 1.2% v/v alcohol.	Review	Until recently the NAB and LAB production techniques were implemented mostly in multinationals, requiring considerable investment in production and marketing, the present dynamics in the market allows the implementation of these methods even on a small scale.<br><br>The post-fermentation techniques focused on the complete or partial removal of the alcohol (ethanol) from the original beer by thermal treatments are falling film evaporation, continuous vacuum rectification, and thin layer evaporation<br><br>Membrane separation processes can be broken down into nanofiltration, reverse osmosis, osmotic distillation, dialysis, and pervaporation.<br>The biological methods of the NAB production based on limited alcohol formation can<br>be divided according to the production equipment they require, such as traditional brewery equipment requirements (changed mashing process, arrested or limited

					fermentation process, cold contact process, and utilization of special yeast) or use of special equipment (continuous limited fermentation).
Wine	Dequin, S., Escudier, J. L., Bely, M., Noble, J., Albertin, W., Masneuf-Pomarede, I., . . . Sablayrolles, J. M. (2017). How to adapt winemaking practices to modified grape composition under climate change conditions. Oeno One, 51(2), 205-214. doi:10.20870/oeno-one.2016.0.0.1584	To review current microbiological and technological strategies to overcome issues related to adaptation of enological practices (linked to alcohol content and the global acidity of wine) in the context of climate change.	Not mentioned – focus on dealcoholisation of wine (lower alcohol content but not no alcohol content)	Review	Several non-genetically modified organism (GMO) strains – *S. cerevisiae* or interspecific hybrids of the *Saccharomyces* genus – have yet been developed using different strategies, and some of them allow decreasing the final ethanol concentration by up to 1%. Several membrane-based technologies have also been developed not only to reduce the ethanol content of wines but also to increase the acidity and more generally to control the wine pH. New strategies are also proposed to improve the control of winemaking, especially the management of alcoholic fermentation of sugar-rich musts and the control of oxidation during the process.
Wine	Liguori, L., Albanese, D., Crescitelli, A., Di Matteo, M., & Russo, P. (2019). Impact of dealcoholization on quality properties in white wine at various alcohol content levels. Journal of Food Science and Technology-Mysore, 56(8), 3707-3720. doi:10.1007/s13197-019-03839-x	To evaluate samples of dealcoholized white wine (cvFalanghina, originally 12.5 vol%) in different alcohol content levels (from 9.8 to 0.3 vol%) dealcoholized through the osmotic distillation process	Tse of denominations ‘‘dealcoholized wine’’ and ‘‘partially dealcoholized wine’’ depends on the legislation of each state. Since 2012, the OIV has adopted two definitions: ‘‘Beverages obtained by wine dealcoholization’’ for those beverages with an alcohol content lower than 0.5 vol%;and ‘‘Beverages obtained by partial wine dealcoholization’’for those in the range of 0.5–8.5 vol%, respectively<br><br>Present study investigates the impact of OD on the evolution of oenological parameters and volatile compounds in white wine at different levels of alcohol, up to a content lower than 0.5 vol%.	Study – dealcoholisation tests	No significant differences in total phenols, flavonoids, organic acids and total acidity were observed among the wine samples at different alcohol content levels.<br>The volatile compounds content decreased with increasing alcohol removal.<br>Results of the sensory evaluation indicated a significant change in terms of acidity, odour, sweetness and body taste in dealcoholized wine (0.3 vol%), giving an overall perceived imbalance and unacceptable taste with respect to the original wine.<br><br>Therefore, in order to balance acid sensation and enhance body and aftertaste, an attempt was made to formulate an alcohol-free wine-based beverage with enhanced odour and sweetness, by adding some floral wine flavours, up to the amount present in the original wine.

	Longo, R., Blackman, J. W., Antalick, G., Torley, P. J., Rogiers, S. Y., & Schmidtke, L. M. (2018). A comparative study of partial dealcoholisation versus early harvest: Effects on wine volatile and sensory profiles. Food Chemistry, 261, 21-29. doi:10.1016/j.foodchem.2018.04.013	To compare different approaches to obtain lower alcohol wine (partial dealcoholisation and early harvest) in terms of volatile and sensory profiles	Lower alcohol wines (broadly defined as containing 5.5–11% v/v alcohol)	Study – dealcoholisation tests	Two Verdelho and Petit Verdot wines were produced from sequential harvests of grapes. The alcohol con-centration of early harvest (EH) and late harvest (LH) wines were respectively 9% and 13.5% v/v for Verdelho, and 10.5% and 13% v/v for Petit Verdot.<br>LH wines were dealcoholised to match the same alcohol level of EH samples using a combined reverse osmosis-evaporative perstraction process.<br>In dealcoholised wines, there was a decrease in volatile compounds (esters particularly) compared to LH treatments.<br>For both varieties, the sensory attribute ratings also decreased following dealcoholisation.<br>Dealcoholised wines were distinctively different from both LH and EH wines even though these wines had similar alcohol level to EH wines.<br>When dealcoholisation is considered for high-alcohol wines, it is important to consider that membrane effects can significantly change depending on the wine non-volatile matrix composition and the level of alcohol reduction required
	Longo, R., Blackman, J. W., Antalick, G., Torley, P. J., Rogiers, S. Y., & Schmidtke, L. M. (2018). Harvesting and blending options for lower alcohol wines: a sensory and chemical investigation. Journal of the Science of Food and Agriculture, 98(1), 33-42. doi:10.1002/jsfa.8434	The aim of this study, performed on Verdelho and Petit Verdot, was to quantify the effectiveness of a monovarietal blend in which wines made from less ripe grapes were blended with an equivalent volume of a wine vinified from riper fruit to produce wines with a lower alcohol content and desirable ripe fruit flavours.	Products are designated as non-alcoholic (0.0% v/v), low alcohol (0.0–5.5% v/v) and lower alcohol (5.5–11.0% v/v) wine, although different countries have inconsistent legislation and terminology.	Study – dealcoholisation tests	11 and 13 attributes, for two wines were selected during sensory descriptive analysis. Intensities of perceived 'acidity', 'sweetness' and 'alcohol' attributes were significantly different between the blend(8.8±0.1% v/v) and mature Verdelho (10.3±0.1% v/v) wines, while no significant differences were found between the PetitVerdot blend (11.0±0.1% v/v) and mature (12.6±0.2% v/v) treatments.<br><br>There was association between sensory descriptors and chemical attributes in the wines, as well as the modifications of sensory and compositional profiles following blending.<br><br>The blending practice described allowed the production of wines with lower alcohol content while retaining similar sensory profiles of the later harvested, riper fruit wines.

	Longo, R., Blackman, J. W., Torley, P. J., Rogiers, S. Y., & Schmidtke, L. M. (2017). Changes in volatile composition and sensory attributes of wines during alcohol content reduction. J Sci Food Agric, 97(1), 8-16. doi:10.1002/jsfa.7757	To identify and summarize possible deleterious influences of the dealcoholisation process and describe best practice strategies to maintain the original wine composition	Not specified	Review	Separation technologies such as membrane filtration, in particular reverse osmosis and evaporative perstraction, in addition to vacuum distillation, represent the most common commercial methods used to produce reduced-alcohol wine.<br><br>However, ethanol removal from wine can result in a significant loss of volatile compounds such as esters (ethyl octanoate, ethyl acetate, isoamylacetate) that contribute positively to the overall perceived aroma.<br><br>These losses can potentially reduce the acceptability of the wine to consumers and decrease their willingness to purchase wines that have had their alcohol level reduced. The change in aroma as a result of the ethanol removal processes is influenced by a number of factors: the type of alcohol reduction process; the chemical-physical properties (volatility, hydrophobicity, sterichindrance) of the aroma compounds; the retention properties of the wine non-volatile matrix; and the ethanol level.
	Ozturk, B., & Anli, E. (2014). Different techniques for reducing alcohol levels in wine: A review. In J. M. Aurand (Ed.), 37th World Congress of Vine and Wine and 12th General Assembly of the Oiv (Vol. 3).	The aim of this review is to provide technical and practical information covering the outstanding techniques that may be used to adjust elevated alcohol concentration in wine and their effect on wine from the point of organoleptic characteristics.	Wine legislation in Turkey: the alcohol content of wine is limited to 9-15% v/v	Review	Several techniques have been developed for the reduction of ethanol content in wines with excessive alcohol content. The techniques at issue are applied on different stages of wine making process. Implementation of different viticulture techniques including reducing leaf area, monitoring maturity and aroma profiles, winemaking practices consist of utilisation of enzymes, different yeast strains and post fermentation practices such as distillation, blending and membrane based systems applications form a basis for the adjustment of elevated alcohol levels in wine
	Varela, C., Dry, P. R., Kutyna, D. R., Francis, I. L., Henschke, P. A., Curtin, C. D., & Chambers, P. J. (2015). Strategies for reducing alcohol concentration in wine. Australian Journal of Grape and Wine Research, 21, 670-679. doi:10.1111/ajgw.12187	This review summarises the latest research aimed at reducing wine alcohol concentrations and discusses the effect of these practices on wine flavour is also discussed	Alcohol concentration in Australian red wines has increased approximately 1% v/v per decade since the1980s. It was rare to encounter wines with more than 14% v/v alcohol a few decades ago; now it is not	Review	High ethanol concentration can reduce the complexity of a wine by suppressing aroma intensity, while also increasing the perception of 'hotness' and 'bitterness'.<br><br>viticultural practices such as decreasing the leaf area to fruit mass (LA/FM) ratio in order to lower sugar concentration in the berry, and

			uncommon to see wines with an alcohol concentration of more than 16% v/v.		harvesting earlier when grapes have lower sugar concentration;<br>• pre-fermentation and winemaking practices, including dilution and blending with, for example, juice from early- harvested low sugar grapes or treatment of grape must to remove sugar;<br>• microbiological strategies, including development of yeast inocula that reduce the efficiency of ethanol production; and<br>• post-fermentation practices and processing technologies, which include blending of high and low alcohol wine, and physical removal of alcohol after fermentation.<br>This<br><br>Strategies aimed at reducing wine alcohol concentration include: viticultural practices, such as decreasing the leaf area to fruit weight ratio in order to lower sugar concen-tration in the berry; pre-fermentation and winemaking practices, including dilution and sugar removal from the grape must; microbiological strategies such as the use of yeasts that are less efficient at ethanol production; and post-fermentation practices and processing technologies, including the physical removal of alcohol after fermentation reviewed
**Beer & Wine**	Mangindaan, D., Khoiruddin, K., & Wenten, I. G. (2018). Beverage dealcoholization processes: Past, present, and future. Trends in Food Science & Technology, 71, 36-45. doi:10.1016/j.tifs.2017.10.018	To discuss dealcoholization processes	Alcohol-free beer (ranging from 0.05 to 1.2% ABV (alcohol by volume)<br>Dealcoholized wine (less than 7% ABV)	Review	The membrane processes demonstrate promising results for beverage dealcoholizations while preserving the sensorial properties.
	Varela, J., & Varela, C. (2019). Microbiological strategies to produce beer and wine with reduced ethanol concentration. Curr Opin Biotechnol, 56, 88-96. doi:10.1016/j.copbio.2018.10.003	To discuss current research on the isolation and/or generation of yeast strains able to produce beer or wine with reduced ethanol concentration, with particular consideration on volatile composition and sensory profile of beer and wine.	low-alcohol beers and alcohol-free beers containing <1.2% v/v and <0.5% v/v of alcohol, respectively.<br><br>Wine not specified	Review	Several yeast-based strategies have been shown to be effective in producing beer and/or wine with reduced ethanol concentration. Regardless of the approach, 'low-ethanol' yeast strains should always be evaluated for sensory impacts they have, ideally, at pilot-scale to determine their real contribution to the flavor characteristics of the final product
**Life cycle assessm ent**	Laoretani, D. S., Sanchez, R. J., Paredes, D. A. F., Iribarren, O. A., & Espinosa, J. (2020). On the conceptual modeling, economic analysis and life cycle	To analyze the economic and environmental performance of three different technologies for the	Alcohol-free beverages (≤0.5% v/v) or beverages with reduced alcohol values (≤3% v/v)	Economic evaluation and life cycle assessment	For each technology it was possible to find operating windows where both economic and

	assessment of partial dealcoholization alternatives of bitter extracts. Separation and Purification Technology, 251. doi:10.1016/j.seppur.2020.117331	on-site concentration of a bitter extract of herbs with simultaneous reduction of its alcoholic content to be able to obtain a beverage without alcohol off-site through the addition of deionized water (nanofiltration, pervaporation, simple distillation).			environmental benefits can be achieved with respect to the base case.<br><br>The best economic and environmental scores were reached by the nanofiltration variant operated at intermediate pressures. The payback period for this alternative was 3 years with an IRR of 31% considering a lifetime of 10 years.
	Margallo, M., Aldaco, R., Barcelo, A., Diban, N., Ortiz, I., & Irabien, A. (2015). Life cycle assessment of technologies for partial dealcoholisation of wines. Sustainable Production and Consumption, 2, 29-39. doi:10.1016/j.spc.2015.07.007	This work assesses and compares the environmental performance of evaporative pertraction, spinning cone column and reverse osmosis using a life cycle assessment methodology based on the use of two variables: natural resources sustainability (NRS) and environmental burdens sustainability (EBS).	The European Com-mission regulation (EC, 2009) has set that in wine dealcoholisation should not remove more than 2 percentage points of ethanol and that the minimum concentration should not be less than 8.5% v/v. Therefore, a small adjustment in the alcohol con-tent between 1 and 2% is currently one of the most important objectives for the wine industry.<br><br>dealcoholised wine (13.26% v/v)	Life cycle assessment	For the technologies, reverse osmosis presented a higher energy consumption and lower materials and water demands than did evaporative pertraction, whereas the latter displayed better results in all of the air categories except for human health effects and in three of the five water categories. The highest consumption of natural resources and the greatest environmental burdens in all of the categories were given by the spinning cone column technology. Nevertheless, this situation was reversed when the wastewater from the spinning cone column process, with its high ethanol content, was energy valorised.
	Paredes, D. A. F., Laoretani, D. S., Morero, B., Sánchez, R. J., Iribarren, O. A., & Espinosa, J. (2020). Screening of membrane technologies in concentration of bitter extracts with simultaneous alcohol recovery: An approach including both economic and environmental issues. *Separation and Purification Technology*, *237*, 116339.	To study concentration of a bitter extract with simultaneous recovery of ethanol via either nanofiltration or pervaporation from economic and environmental standpoints.	Non-alcoholic; i.e., ≤0.5% v/v alcohol	life cycle assessment	The different studied alternatives are ranked from both economic and environmental standpoints.<br><br>Nanofiltration with the membrane NF99 HF (30 °C and 16 bar) showed the best economic and environmental performance.<br><br>On the other hand, pervaporation with PDMS membranes may be a suitable choice whenever the operating conditions allow for heat integration between the retentate and the permeate.

**Alcohol By Volume**	Zendeboodi, F., Jannat, B., Sohrabvandi, S., Khanniri, E., Mortazavian, A. M., Khosravi, K., . . . Esmaeili, S. (2019). Monitoring of ethanol content in non-alcoholic beer stored in different packages under different storage temperatures. Biointerface Research in Applied Chemistry, 9(6), 4624-4628. doi:10.33263/briac96.624628	To determine ethanol production for the studied non-alcoholic beer brands stored at different packages under different storage temperatures	Not mentioned	Four non-alcoholic beer brands (A, B, C and D) were stored for 6 months in three types of packaging material (PET, glass and tin can bottles) at two temperature conditions (4 and 24 C).	All three factors (increase in temperature storage, storage period and PET packaging) had significant effect on ethanol production in non-alcoholic beers, however maximum ethanol concentration (0.240 w/v %) in beers did not exceed of allowed amount (0.5 % V/V) in non-alcoholic beers
**Traditional beverages**	Baschali, A., Tsakalidou, E., Kyriacou, A., Karavasiloglou, N., & Matalas, A. L. (2017). Traditional low-alcoholic and non-alcoholic fermented beverages consumed in European countries: a neglected food group. Nutrition Research Reviews, 30(1), 1-24. doi:10.1017/s0954422416000202	To review the dietary role, nutrient composition, health benefits and other relevant aspects of diverse ethnic low-alcoholic and non-alcoholic fermented beverages consumed by European populations.	The use of the terms 'alcoholic beverage', 'LAFB' and 'NAFB' is subject to varying regulations in different European countries.<br>According to EU Regulation 169/2011 on the provision of Food information to consumers and the European Parliament Resolution 2015/2543 (RSP) an 'alcoholic beverage' contains an 'alcoholic strength by volume' of more than 1·2%,whereas a 'low-alcoholic beverage' refers only to beverages which have an ABV of 1·2 % or less. For the majority of the European countries, the limit of ABV for non-alcoholic beverage is considered 0·5%	Review	A variety of traditional LAFB and NAFB are consumed in European regions, such as kefir, kvass, kombucha and hard aliye.<br><br>Kefir is milk based fermented low alcoholic fermented beverage<br>Boz and kvass are cereal based low alcoholic fermented beverages<br>There are also fruit and vegetables low alcoholic fermented beverages, and herbs, spices and aromatic plant based non and low alcoholic beverages. (e.g. kmbucha, ginger beer, root beer)
**Consumption and purchase**					
	Anderson, P., Jané Llopis, E., O'Donnell, A., Manthey, J., & Rehm, J. (2020). Impact of low and no alcohol beers on purchases of alcohol: interrupted time series analysis of British household shopping data, 2015-2018. Bmj Open, 10(10), e036371. doi:10.1136/bmjopen-2019-036371	To assess the impact of introduction of new low and no alcohol beers and reformulated beers in Great Britain on average alcoholic strength of beer and number of grams of alcohol purchased by households	Definition for analysis:<br>No or low alcohol beer : 3.5%ABV and less	Interrupted time series analysis of introduction of new no and low alcohol beers during 2017–2018 (event 1) and reformulation of existing beers to contain less alcohol during 2018 (event 2)	Overall, the volume of purchases of new low and no alcohol beer products (2.6% of the volume of all beers purchased during 2018) and of new reformulated beer products (6.9% of the volume of all beers purchased during 2018) was very small.<br><br>Interrupted time series analyses found a combined associated impact of both events

				Based on purchase data from Kantar Worldpanel's household shopping panel of 64 286 British households for 2015–2018.	with relative reductions of alcohol by volume of beer between 1.2% and 2.3%; purchases of grams of alcohol within beer between 7.1% and 10.2%; and purchases of grams of alcohol as a whole between 2.6% and 3.9<br><br>The reductions were greater for reformulation than for the introduction of new low and no alcohol products. Reductions were independently higher for younger age groups of shoppers and for households that bought the most alcohol
	Anderson, P., Llopis, E. J., & Rehm, J. (2020). Evaluation of Alcohol Industry Action to Reduce the Harmful Use of Alcohol: Case Study from Great Britain. Alcohol and Alcoholism, 55(4), 424-432. doi:10.1093/alcalc/agaa029	To describe a case study in the British market of one of the global beer-producing companies that has set a target to increase the proportion of its products with an alcohol by volume (ABV) of3.5% or less, and to reduce the mean ABV of its beer products..	Definition for analysis:<br><br>No or low alcohol beer: 3.5%ABV and less	Descriptive statistics and time-series analyses using Kantar Worldpanel's British household purchase data for 2015–2018	15.7% of the company's beer products had an ABV of 3.5% or less in 2018, compared with 8.8% in 2015. The mean ABV of its beer products dropped from 4.69 in 2015 to 4.55 in 2018.<br><br>The changes were associated with reduced purchases of grams of alcohol within its beer products.<br><br>The associated reductions in purchases of alcohol in all beer and in all alcohol products suggest no evidence of overall switching to other higher strength beer or alcohol products
	Kypri, K., Harrison, S., & McCambridge, J. (2020). Ethanol Content in Australian and New Zealand Beer Markets: Exploratory Study Examining Public Health Implications of Official Data and Market Intelligence Reports. Journal of Studies on Alcohol and Drugs, 81(3), 320-330. Retrieved from <Go to ISI>://WOS:000600455400004	To investigate the public health implications of recent developments in beer product availability, marketing, and country-level consumption patterns in terms of ethanol in Australia and New Zealand	Australia:<br><br>Low : less than 3.0%ABV<br>Mid 3.0-3.5% %ABV<br>High: more than 3.5 %ABV<br><br>New Zealand:<br><br>Low: less than 2.5%ABV<br>Mid: 2.5-4.35 %ABV<br>High: 4.351-5.0% ABV<br>Very high: more than 5.0%ABV	Analysis of official data reporting beverage- and strength-specific volumes of ethanol available for sale in beer from 2000 to 2016, a period in which the countries had similar consumption trends; and did a thematic analysis of "market intelligence" reports.	Per capita ethanol beer sales fell in both countries, accompanied by increases in market share of higher %ABV categories. Different definitions of beer strength hampered comparison between countries. In Australia, consumption of ethanol in mid-strength beer (3.01%–3.5%ABV) increased, whereas consumption of low-strength beer (<3%ABV) decreased. In New Zealand, consumption of high-strength beer (4.351%–5%ABV) increased whereas that of traditional mid-strength beer (2.501%–4.35%ABV) decreased substantially.<br><br>Market reports cited consumer health concerns and demand for "craft beer" (typically high-strength) as competing influences in both markets, and reduced-

					alcohol beer as "the alcoholic drinks industry's—potentially lucrative—shield against accusations of irresponsibility.
**Health**					
**Anxiety**	Franco, L., Galan, C., Bravo, R., Bejarano, I., Penas-Lledo, E., Rodriguez, A. B., . . . Cubero, J. (2015). Effect of non-alcohol beer on anxiety: Relationship of 5-HIAA. Neurochemical Journal, 9(2), 149-152. doi:10.1134/s181971241502004x	To analyze the possible anxiolytic effect of non-alcoholic beer on neuroendocrine levels of cortisol, melatonin and serotonin, in a population under stress.	Non-alcohol beer (Mahou Laiker Without®)	A healthy student population (N=16) was enrolled during exam period. They consumed 1 nonalcoholic beer (330 mL) at dinner time for 14 nights. Melatonin, serotonin and cortisol levels were measured by quantifying their early morning urinary metabolites collected weekly	The consumption of non-alcoholic beer significantly reduced significantly the nocturnal serotonin levels whereas there were no substantial changes on melatonin and cortisol levels.<br>Anxiety/State parameters decreased after consumption of non-alcoholic beer at dinner time regarding to control values.
**Bone loss**	Yin, J., Winzenberg, T., Quinn, S., Giles, G., & Jones, G. (2011). Beverage-specific alcohol intake and bone loss in older men and women: a longitudinal study. European Journal of Clinical Nutrition, 65(4), 526-532. doi:10.1038/ejcn.2011.9	To describe the associations between total and beverage-specific alcohol intake and bone loss in older men and women.	Low alcohol beer Not mentioned	Observational: total of 862 randomly selected subjects (mean age 63 years, range 51–81, 51% men) were studied at baseline and 2 years later.	In women, the frequency of drinking low alcohol beer was positively associated with bone mass density at the lumbar spine but not at hip.
**Diabetes**	Mateo-Gallego, R., Perez-Calahorra, S., Lamiquiz-Moneo, I., Marco-Benedi, V., Bea, A. M., Fumanal, A. J., . . . Civeira, F. (2020). Effect of an alcohol-free beer enriched with isomaltulose and a resistant dextrin on insulin resistance in diabetic patients with overweight or obesity. Clinical Nutrition, 39(2), 475-483. doi:10.1016/j.clnu.2019.02.025	To explore the effect of an alcohol-free beer with modified carbohydrate composition almost completely eliminating maltose and adding isomaltulose (16.5 g/day)and a resistant maltodextrin (5.28 g/day) in comparison to a regular alcohol-free beer on glycemiccontrol of diabetic subjects with overweight or obesity	Alcohol-free beer (no further specification)	Subjects (N=41) randomized into two groups: a) consumption of 66 cL/day of regular alcohol-free beer for the first 10 weeks and 66 cL/day of alcohol-free beer with modified carbohydrate composition for the next 10 weeks; b) the same described intervention in opposite order	An alcohol-free beer including the substitution of regular carbohydrates for low doses of isomaltulose and the addition of a resistant maltodextrin within meals led to an improvement in insulin resistance in subjects with T2Diabetes Mellitus and overweight or obesity
**Cardiovascular**	Daimiel, L., Mico, V., Diez-Ricote, L., Ruiz-Valderrey, P., Istas, G., Rodriguez-Mateos, A., & Ordovas, J. M. (2021). Alcoholic and Non-Alcoholic Beer Modulate Plasma and Macrophage microRNAs Differently in a Pilot Intervention in Humans with Cardiovascular Risk. Nutrients, 13(1). doi:10.3390/nu13010069	To test if beer and non-alcoholic beer consumption modify the levels of a panel of 53 cardiometabolic microRNAs in plasma and macrophages, and to analyse whether this modulation was associated with CVD risk and discern the molecular processes affected by such modulation in subjects with high CVD risk.	Non-alcoholic beer (no further specification)	Seven non-smoker men aged 30–65 with high cardiovascular risk were recruited for a non-randomised cross-over intervention consisting of the ingestion of 500 mL/day of beer or non-alcoholic beer for 14 days with a 7-day washout period between interventions	Non-alcoholic beer intake modulated differentially plasma and macrophage microRNAs. Specifically, microRNAs related to inflammation increased after beer consumption and decreased after non-alcoholic beer consumption
	Noguer, M. A., Cerezo, A. B., Donoso Navarro, E., & Garcia-Parrilla, M. C. (2012). Intake of alcohol-free red wine	To evaluate whether alcohol-free wine has any effect on antioxidant enzymes.	Alcohol-free wine (no further specification)	Randomized cross-over human intervention. (N=8)	Increase in the activity of the antioxidant enzymes is not due to the alcohol content in wine but to the polyphenolic composition.

	modulates antioxidant enzyme activities in a human intervention study. Pharmacol Res, 65(6), 609-614. doi:10.1016/j.phrs.2012.03.003			A low phenolic diet (LPD) was designed to prevent interference from polyphenols in other food sources. In the first period, the volunteers ate only this low phenolic diet; in the second, they ate this diet and also drank 300 mL of alcohol-free wine. The enzymes under study were: superoxide dismutase, catalase, glutathione peroxidase and glutathione reductase.	
	Chiva-Blanch, G., Magraner, E., Condines, X., Valderas-Martínez, P., Roth, I., Arranz, S., . . . Estruch, R. (2015). Effects of alcohol and polyphenols from beer on atherosclerotic biomarkers in high cardiovascular risk men: a randomized feeding trial. Nutr Metab Cardiovasc Dis, 25(1), 36-45. doi:10.1016/j.numecd.2014.07.008	To evaluate the effects of ethanol and the phenolic compounds of beer on classical and novel cardiovascular risk factors.	containing <1 g of ethanol and 1243 mg of total polyphenols	Thirty-three high risk male volunteers were included in a randomized, crossover feeding trial. After a washout period, all subjects received beer (30 g alcohol/d, 660 mL), the equivalent amount of polyphenols as non-alcoholic beer (990 mL), and gin (30 g alcohol/d, 100 mL) for 4 weeks. All outcomes were evaluated before and after each intervention period.	Non-alcoholic beer decreased systolic blood pressure and increases plasma folic acid (but not other tested markers)
	Chiva-Blanch, G., Condines, X., Magraner, E., Roth, I., Valderas-Martínez, P., Arranz, S., . . . Estruch, R. (2014). The non-alcoholic fraction of beer increases stromal cell derived factor 1 and the number of circulating endothelial progenitor cells in high cardiovascular risk subjects: a randomized clinical trial. Atherosclerosis, 233(2), 518-524. doi:10.1016/j.atherosclerosis.2013.12.048	To compare the effects of moderate consumption of beer, non-alcoholic beer and gin on the number of circulating endothelial progenitor cells (EPC) and EPC-mobilizing factors.	Non-alcoholic beer:<0.1%,ABV	Crossover trial (N=33) men at high cardiovascular risk were randomized to receive beer (30 g alcohol/d), the equivalent amount of polyphenols in the form of non-alcoholic beer, or gin (30 g alcohol/d) for 4 weeks. Diet and physical exercise were carefully monitored.	Non-alcoholic beer increased number of circulating enothelian progenitor cells, and stromal cell derived factor1 in high cardoivascular risk men
**Lactation**	Codoner-Franch, P., Hernandez-Aguilar, M. T., Navarro-Ruiz, A., Lopez-Jaen, A. B., Borja-Herrero, C., & Valls-Belles, V. (2013). Diet Supplementation During Early	To test whether supplementing the diet of breastfeeding mothers with non-alcoholic beer, a product rich in antioxidants, could improve their oxidative status	Non-alcoholic beer (not specified)	A prospective clinical study of breastfeeding mothers (N=60) and their infants were allocated to either a control group(n=30) on a free	Results indicate a positive effect of non-alcoholic beer supplementation on oxidative stress in mothers. No difference in oxidant markers was found in the infant's urine.

	Lactation with Non-alcoholic Beer Increases the Antioxidant Properties of Breastmilk and Decreases the Oxidative Damage in Breastfeeding Mothers. Breastfeeding Medicine, 8(2), 164-169. doi:10.1089/bfm.2012.0059	and the antioxidant content of their milk.		diet or a study group (n=30) on a free diet supplemented with 660 mL of non-alcoholic beer/day.	
	Schneider, C., Thierauf, A., Kempf, J., & Auwarter, V. (2013). Ethanol Concentration in Breastmilk After the Consumption of Non-alcoholic Beer. Breastfeeding Medicine, 8(3), 291-293. doi:10.1089/bfm.2012.0156	To determine how much of ethanol in non-alcoholic beer drank by the mother may reach the breastfed child.	According to regulations in the United States and most European countries, these ''alcohol-free'' beverages may still contain ethanol up to 1.2% by volume	Healthy breastfeeding women (N=15) participated in the study - they were asked to drink 1.5 L of non- alcoholic beer within 1 hour. Breastmilk samples were collected immediately after the end of drinking as well as 1 and 3 hours later and analysed for ethanol presence.	In two women, trace amounts of ethanol (up to 0.0021 g/L) were found in the samples gained immediately after the drinking period. In the other samples ethanol could not be detected .
**Liver**	Macias-Rodriguez, R. U., Ruiz-Margain, A., Roman-Calleja, B. M., Espin-Nasser, M. E., Flores-Garcia, N. C., Torre, A., . . . Lozano-Cruz, O. A. (2020). Effect of non-alcoholic beer, diet and exercise on endothelial function, nutrition and quality of life in patients with cirrhosis. World Journal of Hepatology, 12(12), 1299-1313. doi:10.4254/wjh.v12.i12.1299	To evaluate the effect of diet + exercise and non-alcoholic beer on nutritional status, endothelial function and quality of life in patients with cirrhosis.	Non-alcoholic beer (not specified)	Randomized open clinical trial (N=43). The intervention (non-alcoholic beer + diet + exercise) and control (water + diet + exercise) group. Treatment consisted of 330 mL non-alcoholic beer/day or the same amount of water, plus an individualized dietary plan and an exercise program.	All measured nutritional parameters improved in the intervention group, compared to only 2 in the control group. Quality of life improved in both groups; however, more domains improved in the intervention group
**Sleep**	Franco, L., Sánchez, C., Bravo, R., Rodríguez, A. B., Barriga, C., Romero, E., & Cubero, J. (2012). The sedative effect of non-alcoholic beer in healthy female nurses. Plos One, 7(7), e37290. doi:10.1371/journal.pone.0037290	To analyze the sedative effect of hops as a component of non-alcoholic beer on the sleep/wake rhythm in a work-stressed population	0,0% alcohol	Within subjects experiment conducted with healthy female nurses (n = 17) working rotating and/or night shifts. Overnight sleep and chronobiological parameters were assessed by actigraphy after moderate ingestion of non-alcoholic beer containing hops with supper for 14 days (treatment). Data were obtained in comparison with own control group without consumption of beer during supper.	Sleep latency diminished in the Treatment group when compared to the Control group, also total activity<br>Anxiety decreased in treatment group
	Franco, L., Bravo, R., Galan, C., Rodriguez, A. B., Barriga, C., & Cubero, J. (201). Effect of non-alcoholic beer on Subjective Sleep Quality in a university stressed population. Acta Physiologica Hungarica, 101(3), 353-361. doi:10.1556/APhysiol.101.2014.3.10	To determine the impact of non-alcoholic beer on subjective sleep quality using the Pittsburgh Sleep Quality Index (PSQI)	Alcohol-free beer (MahouLaiker Without)	University students (N=30). The study took place during a period of 3 weeks, the first 7 days were used for the Control, and during the following 14 days the students	The results revealed that Subjective Sleep Quality improved and Sleep Latency decreased in the case of those students who drank one beer during dinner compared to the Control.<br>The overall rating Global Score of Quality of Sleep also improved significantly.

				ingested non-alcoholic beer during dinner.	
Sport	Scherr, J., Nieman, D. C., Schuster, T., Habermann, J., Rank, M., Braun, S., ... & Halle, M. (2012). Nonalcoholic beer reduces inflammation and incidence of respiratory tract illness. *Medicine & Science in Sports & Exercise*, *44*(1), 18-26.	To determine whether ingestion of non-alcoholic beer polyphenols for 3 weeks before and 2 weeks after a marathon would attenuate postrace inflammation and decrease upper respiratory tract illness incidence	ERDINGER Alkoholfrei	Healthy male runners (N= 277, age = 42.9 yr) were randomly assigned to 1–1.5 dl of NAB or placebo (PL) beverage(double-blind design) for 3 weeks before and 2 weeks after the Munich Marathon. Blood samples were collected and analyzed for inflammation measures (interleukin-6 and total blood leukocyte counts). URTI rates, assessed by the Wisconsin Upper Respiratory Symptom Survey, were compared between groups during the 2-wk periodafter the race	Consumption of 1–1.5 LIdj1of NAB around the marathon competition reduces postrace inflammation .<br>Continued ingestion of the nonalcoholic beer during the 2-wk period after the race reduced the incidence of clinically relevant URTI.<br><br>Not clear whether these findings are linked and dependent on each other
Sport	Castro-Sepulveda, M., Johannsen, N., Astudillo, S., Jorquera, C., Alvarez, C., Zbinden-Foncea, H., & Ramirez-Campillo, R. (2016). Effects of Beer, Non-Alcoholic Beer and Water Consumption before Exercise on Fluid and Electrolyte Homeostasis in Athletes. Nutrients, 8(6). doi:10.3390/nu8060345	To compare the effect of beer, non-alcoholic beer and water consumption before exercise on fluid and electrolyte homeostasis - on urine volume, sweat rate, evaporative water loss, plasma electrolytes (Na+and K+), and USG in young athletes.	Non-alcoholic beer (0% alcohol)	Double-blind, randomized study (N=7)<br>Seven male soccer players performed 45 min of treadmill running at 65% of the maximal heart rate, 45 min after ingesting 0.7 L of water (W), beer (AB) or non-alcoholic beer (NAB).	After exercise, body mass decreased in W (′1.1%), AB (′1.0%) and NAB (′1.0%).<br><br>In the last minutes of exercise, plasma Na+was reduced in W (′3.9%) and AB (′3.7%),plasma K+was increased ($p< 0.05$) in AB (8.5%), and USG was reduced in W (′0.9%) and NAB(′1.0%).<br><br>Collectively, these results suggest that non-alcoholic beer before exercise could help maintain electrolyte homeostasis during exercise
	Desbrow, B., Murray, D., & Leveritt, M. (2013). Beer as a Sports Drink? Manipulating Beer's Ingredients to Replace Lost Fluid. International Journal of Sport Nutrition and Exercise Metabolism, 23(6), 593-600. doi:10.1123/ijsnem.23.6.593	To investigate the effect of manipulating the alcohol and sodium content of beer on fluid restoration following exercise	Low alcohol beer (2.3% ABV)	Seven male volunteers exercised on a cycle ergometer until 1.96 ± 0.25% body mass (mean± SD) was lost. Experimental trials consisted of exercise-induced mass loss (target 2.0% body mass) followed by consumption of a test beverage. Drinks included a low-alcohol beer (2.3% ABV; LightBeer), a low-alcohol beer with 25 mmol×L–1 of added sodium (LightBeer+25), a full-strength beer (4.8% ABV;	Significantly enhanced net fluid balance was achieved following the LightBeer+25 trial (–1.02 ± 0.35 kg) compared with the Beer (–1.59 ± 0.32 kg) and Beer+25 (–1.64 ± 0.28 kg) treatments.<br><br>Accumulated urine output was significantly lower in the LightBeer+25 trial (1477 ± 485 ml) compared with the Beer+25 (2101 ± 482 ml) and Beer (2175 ± 372 ml) trials.<br><br>A low alcohol beer with added sodium offers a potential compromise between a beverage with high social acceptance and one which

				Beer), or a full-strength beer with 25 mmol×L–1 of added sodium (Beer+25). Measures of net fluid balance, urine production, breath alcohol concentration, and subjective ratings of gastrointestinal tolerance were collected hourly as dependent variables across a subsequent 4h rest period	avoids the exacerbated fluid losses observed when consuming full strength beer
	Desbrow, B., Cecchin, D., Jones, A., Grant, G., Irwin, C., & Leveritt, M. (2015). Manipulations to the Alcohol and Sodium Content of Beer for Postexercise Rehydration. International Journal of Sport Nutrition and Exercise Metabolism, 25(3), 262-270. doi:10.1123/ijsnem.2014-0064	To explore the effect of further manipulations to the alcohol and sodium content of beer on fluid restoration following exercise	Low alcohol beer (2.3% ABV)<br>Midstrength beer (3.5% ABV)	Twelve male volunteers lost 2.03 ± 0.19% body mass (mean ± SD) using cycling-based exercise. Participants were then randomly allocated a different beer to consume on four separate occasions. Drinks included low alcohol beer with 25 mmol·L-1 of added sodium [LightBeer+25], low alcohol beer with 50 mmol·L-1 of added sodium [LightBeer+50], midstrength beer (3.5% ABV) [Mid] or midstrength beer with 25 mmol·L-1 of added sodium [Mid+25]. Body mass, urine samples and regulatory hormones were obtained before and 4 hr after beverage consumption	The principle finding indicates that the greatest fluid retention occurred with the consumption of beer containing the highest electrolyte content combined with the lowest concentration of alcohol.
	Krennhuber, K., Kahr, H., & Jager, A. (2016). Suitability of Beer as an Alternative to Classical Fitness Drinks. Current Research in Nutrition and Food Science, 4, 26-31. doi:10.12944/CRNFSJ.4.Special-Issue-October.04	To compare the chemical and physical properties of different beers, alcoholic (A), alcohol-free (F) and yeast-clouded alcohol-free beer (Y) with commercially available fitness drinks (I) labelled isotonic and mineral nutrient containing to show the suitability of beer as a fitness drink.	Non alcoholic beers: Clausthaler Classic, Becks alcohol free, Erdinger alcohol free, Weihenstephaner Hefeweißbier alcohol free, Schneider Weiße alcohol free, Paulaner Hefeweißbier alcohol free.	Different beers, alcoholic, alcohol-free and yeast-clouded, alcohol-free beer were analyzed by High Performance Liquid Chromatography and Ion chromatography to determine the content of mineral salts and carbohydrates.	The proposition that beer in general is an appropriate fitness drink could not be confirmed. None of the beverages, even the labeled fitness drinks, matches the E.C. recommendation for sodium content. Alcoholic beer, besides the negative effects of the alcohol, is highly hypertonic. Clear alcohol-free beer has a lack of mineral nutrients.<br><br>According to Austrian law, most yeast clouded alcohol-free beer might be declared

				Different analyses were performed to obtain the characteristics of the beverages and subsequently to calculate the osmolality for comparison of the results with the EC recommendations and Austrian declaration laws	and promoted as isotonic, mineral nutrient containing, as it matches the Codex Alimentarius threshold values.
	Wijnen, A. H., Steennis, J., Catoire, M., Wardenaar, F. C., & Mensink, M. (2016). Post-Exercise Rehydration: Effect of Consumption of Beer with Varying Alcohol Content on Fluid Balance after Mild Dehydration. Front Nutr, 3, 45. doi:10.3389/fnut.2016.00045	To investigate the effect of beer consumption, with varying alcohol content, on fluid balance after exercise-induced dehydration	Non-alcoholic beer (0.0%), low-alcohol beer (2.0%),	Randomized crossover design (N=11) Subjects exercised on a cycle ergometer for 45 min at 60% of their maximal power output (Wmax) until mild dehydration (1% body mass loss). After, in random order, one of five experimental beverages was consumed, in an amount equal to 100% of their sweat loss: non-alcoholic beer (0.0%), low-alcohol beer (2.0%), full-strength beer (5.0%), an isotonic sports drink, and water. Fluid balance was assessed up till 5 h after rehydration	There was no difference in net fluid balance between the different beverages. Only a short-lived difference between full-strength beer and the isotonic sports drink in urine output and NFB was observed after mild exercise-induced dehydration. Fluid replacement – either in the form of non-alcoholic beer, low-alcoholic beer, full-strength beer, water, or an isotonic sports drink of 100% of body mass loss was not sufficient to achieve full rehydration.
**Review**	Hagemann, M. H., Schmidt-Cotta, V., Marchioni, E., & Braun, S. (2017). Chance for Dry-hopped Non-alcoholic Beverages? Part 2: Health Properties and Target Consumers. Brewing Science, 70(7-8), 118-123. doi:10.23763/BrSc17-10Hagemann	To review the main potential health beneficial properties of alcohol-free dry-hopped beer and dry-hopped relaxation drink, and describe the main target consumer groups.	Not mentioned	Review	AFB beer is seen as a substitution for conventional beer, e.g. for people who like the taste of beer but need to operate vehicles, are pregnant or have a medical condition that does not allow the consumption of alcohol, use medication that might interact with the ethanol of conventional beer, belong to religious or social groups that agreed on alcohol-abstinence, or have concerns about the negative health aspects of alcohol-consumption<br><br>Alcohol-free beer and functional drinks are two strongly growing markets within the beverage sector. The success of alcohol-free beer and functional drinks with relaxing, stress reducing properties might be largely based on their health-beneficial properties. Additionally, exotic flavors along with a high product quality are also key value propositions of these products.

	Mellor, D. D., Hanna-Khalil, B., & Carson, R. (2020). A review of the potential health benefits of low alcohol and alcohol-free beer: Effects of ingredients and craft brewing processes on potentially bioactive metabolites. *Beverages*, *6*(2), 25.	To review the potential health benefits of low alcohol and alcohol-free beer.	Not mentioned	Review (narrative – no methodology)	There is potential to enhance the bioactive qualities of beer whilst reducing the alcohol and energy content through novel brewing approaches often used in craft brewing, in terms of ingredients, brewing methods and type of fermentation. Consumer demand to produce a greater variety of beer types, including alcohol-free beers, may also help to increase the number of beers which may have greater potential to improve health, with lower levels of alcohol, while still being tasty products. As low alcohol, prebiotic and bioactive containing beers are developed, it is important that their potential health benefits and risks are fully assessed
**Perception and preferences**					
**Topic**	**Reference**	**Focus of the paper**	**Definition of no and low alc product**	**Methods**	**Key findings**
	Blackmore, H., Hidrio, C., Godineau, P., & Yeomans, M. R. (2020). The effect of implicit and explicit extrinsic cues on hedonic and sensory expectations in the context of beer. Food Quality and Preference, 81. doi:10.1016/j.foodqual.2019.103855	To investigate the role of extrinsic cues in generating sensory and hedonic expectations of beer.	Non alcoholic beer :0.0%ABV<br>3.0%ABV – different volumes	Repeated measures, 4 experiments, online survey (N=166 across experiments)	While the alcohol content affected participants' sensory expectations, it had no significant effect on expected liking.<br><br>With the addition of more explicit information (alcohol content) to the label, the effect of label colour was diminished<br><br>When information about alcohol content was added, the effect of colour on expectations decreased<br><br>Describing sensory qualities of beer using a sensory descriptor had a larger effect than labelled alcohol content and label colour.<br><br>Overall, consumer expectations were mostly influenced by sensory descriptor, followed by labelled alcohol content, with the effect of label colour
	Frost, R., Quinones, I., Veldhuizen, M., Alava, J. I., Small, D., & Carreiras, M. (2015). What Can the Brain Teach Us	To compare reactions of human subjects to different types of wine using functional magnetic	Wine	FMRI study (N=26)	Contrary to expectation, significantly greater activation was found for low-alcohol than for high-alcohol content wines in brain regions

	about Winemaking? An fMRI Study of Alcohol Level Preferences. Plos One, 10(3). doi:10.1371/journal.pone.0119220	imaging, focusing on brain regions critical for flavor processing and food reward	(Low alcohol contents: between 13 and 13.5%; High alcohol contents: be-tween 14.5 and 15.0%)		that are sensitive to taste intensity, including the insula as well as the cerebellum. Authors interpret the preferential response to the low-alcohol content wines as arising from top-down modulation due to the low alcohol content wines inducing greater attentional exploration of aromas and flavours.
	Fukuda, M. (2019). The effects of non-alcoholic beer on response inhibition: An open-label study. Learning and Motivation, 66, 46-54. doi:10.1016/j.lmot.2019.04.002	To test the hypothesis that the taste, smell, and sight of non-alcoholic beer (alcohol cue) will induce a conditioned response even when participants know that the beer is non-alcoholic.	0.00% Non-alcoholic beer	Three experimental studies (N=32/21/26)	The results suggest that non-alcoholic beer impairs inhibitory responses (i.e.,produces a conditioned response) in older drinkers because these drinkers more strongly associate the alcohol conditioned stimulus with the unconditioned stimulus than do younger participants.
	Smeets, P. A. M., & de Graaf, C. (2019). Brain Responses to Anticipation and Consumption of Beer with and without Alcohol. Chemical Senses, 44(1), 51-60. doi:10.1093/chemse/bjy071	To investigate whether oral exposure to NA-beer with or without alcohol elicits similar brain responses in reward-related areas in a context where regular alcoholic beer is expected.	Non-alcoholic beer (Amstel 0.0, 0.0% alcohol)	FMRI study (N=21) of beer drinkers	No differences were found in acute brain reward upon consumption of NA-beer with and without alcohol, when presented in a context where regular alcoholic beer is expected. This suggests that in regular consumers, beer flavor rather than the presence of alcohol is the main driver of the consumption experience.
	Bucher, T., Deroover, K., & Stockley, C. (2018). Low-Alcohol Wine: A Narrative Review on Consumer Perception and Behaviour. Beverages, 4(4). doi:10.3390/beverages4040082	To provide an introduction on low-alcohol wine, and to provide an overview of the literature on research that investigated perception and behaviour related to low-alcohol wine consumption.	Classification of wines with reduced alcohol content:<br><br>De-alcoholised: <0.5%<br><br>Low alcohol: 0.5–1.2%<br><br>Reduced alcohol: 1.2–4.5%<br><br>Lower alcohol: 5.5–10.5%<br><br>This classification is, however, not explicit and may vary between countries and the applicable legislations.	Review (Narrative)	Taste has been described as another motive for not consuming low-alcohol beverage<br><br>Publicly available peer-reviewed scientific evidence on global patterns and attitudes and consumption behaviours related to low-alcohol wine are currently scarce, a shift in the market is evident.<br><br>Research that investigated consumer behaviour on an individual level across the US, China, Germany, and the UK suggested that mainly the younger generation showed interest in lower alcohol wines<br><br>Experimental studies have shown that a claim on the label about a reduced alcohol content

					reduces appeal of the product and negatively impacts the expected quality<br><br>Total amount of drink consumed increased as the alcohol strength on the label decreased<br><br>The terminology used on the label (e.g., low alcohol, de-alcoholised, reduced alcohol or light) may evoke different consumer perceptions and behaviours
	Bucher, T., Frey, E., Wilczynska, M., Deroover, K., & Dohle, S. (2020). Consumer perception and behaviour related to low-alcohol wine: do people overcompensate? Public Health Nutrition, 23(11), 1939-1947. doi:10.1017/s1368980019005238	To investigate how people evaluate low-alcohol wine (Sauvignon Blanc) and if the reduction in alcohol and the information that a wine is low in alcohol influences consumption.	Wine:<br><br>'new white wine'(12·5 % alcohol content),<br><br>'new low-alcohol white wine'(8·0 % alcohol content)	Randomised controlled trial (N=90)	Mean comparisons showed similar ratings for the low-alcohol condition sand the standard alcohol condition. The mean consumed amount across all conditions did not differ, hence participants who tasted the low-alcohol wine consumed approximately 30 % less alcohol.<br><br>However, participants were willing to pay more for the normal wine compared with the low-alcohol wine.
	Chrysochou, P. (2014). Drink to get drunk or stay healthy? Exploring consumers' perceptions, motives and preferences for light beer. Food Quality and Preference, 31, 156-163. doi:10.1016/j.foodqual.2013.08.006	To investigate consumers' perceptions, motives and preferences for low-alcohol beverages, using light beer as an exemplary case.	Beer<br><br>In terms of alcohol content, the definition of light beer varies across countries. In the US, a beer is considered and marketed as light when the alcohol by volume (ABV) is 4.2%;<br><br>In Australia the ABV must be between 2.2% and 3.2%;<br><br>in Canada the ABV should be between 2.6% and 4.0%.<br><br>These variations often result from country-specific alcohol policies that permit retailstores to sell alcoholic beverages that do not exceed	Online survey using Best-Worst Scaling (N=328) to measure preferences, perceptions and motive also measured	The findings show that light beer is perceived as healthier than regular beer, while the most important motives behind purchase are taste, health and weight management.<br><br>Light beer is further perceived as less tasty, but at the same time healthier, than regular beer,<br><br>Motives related to the low calorie content are more important than motives related to the low alcohol content.<br><br>Preferences for light beer are driven mostly by taste, prior experience and brand.

			a certain per-centage of alcohol content<br><br>In Iceland, light beer is that which has less than 4.5% AB		
	Naspetti, S., Alberti, F., Mozzon, M., Zingaretti, S., & Zanoli, R. (2020). Effect of information on consumer preferences and willingness-to-pay for sparkling mock wines. British Food Journal, 122(8), 2621-2638. doi:10.1108/bfj-06-2019-0469	To explore the role of information about alcohol content, organic labelling and packaging on consumer preferences and willingness-to-pay (WTP) of non-alcoholic sparkling mock wines.	Wine<br><br>Sparkling mock wines (no-alcohol) – alcohol free, 0.0%	In a two-step study, the consumer's expectations and overall liking of two novel brands of mock wines were investigated by focus groups followed by a common hedonic test combined with a choice experiment aimed at measuring consumer willingness to pay. A total of 240 consumers were assigned to two tasting groups of equal size: all were presented at least one brand of mock wine, while drinkers also tasted a familiar brand of low-alcohol sweet sparkling wine. A paper-and-pencil choice experiment followed the tasting sessions.	The results demonstrate that participants in blind or manipulated "informed" conditions are notable to discriminate among mock wines and wine, whereas significant differences in preferences for brands under investigation appeared when labels and other information were disclosed.<br><br>Drinkers and non-drinkers did not differ in hedonic scores of mock wines. While younger participants exhibited the highest scores in blind liking, the overall expected liking is significantly higher for non-drinkers and women if compared, respectively, to drinkers and men.<br><br>WTP for mock wines is influenced by taste, glass bottle packaging and the organic label, while mock-wine colour is not relevant.
	Paixão, J. A., Tavares Filho, E., & Bolini, H. M. A. (2020). Investigation of Alcohol Factor Influence in Quantitative Descriptive Analysis and in the Time-Intensity Profile of Alcoholic and Non-Alcoholic Commercial Pilsen Beers Samples. *Beverages*, *6*(4), 73.	To identify the influence of the alcoholic factor in sensory profile through quantitative descriptive analysis and time intensity analysis of Pilsen beer samples.	Non-alcoholic beer (not further specified)	An acceptance test with 120 consumers of beer was performed. A quantitative descriptive analysis was carried out by 11 trained assessors to determine the sensory profile of beers.	In relation to QDA, significant differences, mainly in relation to the attributes alcoholic aroma and alcoholic flavor, were found between the alcoholic and non-alcoholic samples.<br><br>The correlation between the quantitative descriptive analysis and acceptance tests shown that the alcoholic aroma and the alcoholic flavor contributed as a positive preference driver for the acceptance of the product.<br><br>In addition, the external preference map demonstrated that the samples most

					preferred by consumers are those containing the alcohol in its formulation
	Silva, A. P., Jager, G., van Bommel, R., van Zyl, H., Voss, H. P., Hogg, T., . . . de Graaf, C. (2016). Functional or emotional? How Dutch and Portuguese conceptualise beer, wine and non-alcoholic beer consumption. Food Quality and Preference, 49, 54-65. doi:10.1016/j.foodqual.2015.11.007	To explore conceptualisations, i.e. functional and emotional associations that consumers have with foods/beverages, to understand how NAB consumption is perceived, and compared to beer and wine conceptualisations in the Netherlands and Portugal	Non-alcoholic beer (not further specified)	Qualitative study was per-formed using a focus group approach with moderate consumers of both countries (n= 56).	The study showed similar conceptualisations of the beverages in both countries. NAB has a limited conceptual content, which is mostly functional as a substitute. NAB evokes neutral and negative emotional responses, such as rational conscious and disappointed.
	Silva, A. P., Jager, G., Van Zyl, H., Voss, H. P., Pintado, M., Hogg, T., & De Graaf, C. (2017). Cheers, proost, saúde: Cultural, contextual and psychological factors of wine and beer consumption in Portugal and in the Netherlands. Crit Rev Food Sci Nutr, 57(7), 1340-1349. doi:10.1080/10408398.2014.969396	To identify determinants for consumption of wine, beer, and NAB, using data on consumption patterns from Portugal and the Netherlands	Non-alcoholic beer - beer can be called non-alcoholic depending on the legal definitions, which can vary between countries.<br><br>In Portugal NAB must contain less than 0.5% alcohol by volume (Portaria n. 1/96), whereas in the Netherlands up to 0.1% alcohol by volume is permitted	Review	Wine is perceived as the healthiest beverage, followed by NAB, and regular beer. Motivation for consumption is related to context: wine for special occasions, beer for informal occasions, and NAB for occasions when alcohol is not convenient.<br><br>NAB it is a relatively new beverage, with no cultural roots and consumption is minimal in both countries compared to wine or beer.
	Silva, A. P., Jager, G., Voss, H. P., van Zyl, H., Hogg, T., Pintado, M., & de Graaf, C. (2017). What's in a name? The effect of congruent and incongruent product names on liking and emotions when consuming beer or non-alcoholic beer in a bar (vol 55, pg 58, 2017). Food Quality and Preference, 61, 78-78. doi:10.1016/j.foodqual.2017.03.003	To identify expectations, liking and emotions related to the consumption of conventional beer and non-alcoholic beer (NAB).	Non-alcoholic beer (not further specified)	The labelling of a sample as beer or non-alcoholic beer was employed as a prompt to study the effects on liking and emotions provoked, when drinking a beer or a NAB, in a bar. Over 4 sessions, 155 moderate beer consumers drank a glass of beer or NAB under two different conditions, labelled either correctly or incorrectly with respect to the actual composition of the sample. Questionnaires were used to rate the liking and emotions	The naming of NAB as beer significantly increased the liking and changed one emotion towards a positive direction, namely participants felt more fulfilled.<br><br>When beer was presented as NAB it did not affect the liking but did significantly reduce the intensity of six positive emotions. Participants felt less comforted, exuberant, good, happy, joyful and loving.

				prior to and after consumption.	
	Stanco, M., Lerro, M., & Marotta, G. (2020). Consumers' Preferences for Wine Attributes: A Best-Worst Scaling Analysis. Sustainability, 12(7). doi:10.3390/su12072819	To explore consumers' preferences for wine attributes related to tradition, sustainability, and innovation.	Alcohol-free wine	Regular wine consumers (N=149) from Italy participated in an online survey using Best-Worst Scaling experiment with 11 wine attributes drawn from the literature.	The result shows that the wine attributes more important for consumers, are mainly related to tradition and sustainability. The most important attributes for consumers when purchasing wine are "geographical indications","grape variety", "sustainable certification", "vintage", and "price". By contrast, the innovative wine attributes investigated were ranked among the least important ones, namely "canned wine","alcohol-free wine", and "vegan wine".
	Vasiljevic, M., Couturier, D. L., & Marteau, T. M. (2019). What are the perceived target groups and occasions for wines and beers labelled with verbal and numerical descriptors of lower alcohol strength? An experimental study. Bmj Open, 9(6). doi:10.1136/bmjopen-2018-024412	To assess what a sample of UK weekly drinkers perceived to be the target groups and occasions for drinking wines and beers labelled with different verbal and numerical descriptors of lower alcohol strength.	Focus of the study<br><br>Wine and beer	3390 adults (1697 wine and 1693 beer drinkers) were sampled from a nationally representative UK panel, and participated in a between-subjects experiment in which participants were randomised to 1 of 18 groups with one of three levels of verbal descriptor (Low vs. Super Low vs. No verbal descriptor) and six levels of %ABV (five levels varying for wine and beer, and no level given<br><br>he study gauged participants' perceptions of the type of person that would find the randomised beverage appealing and the type of occasion on which the beverage is likely to be drunk at	A principal component analysis showed that participants perceived pregnant women, sportspeople and those aged 6–13 years old were the target groups for products labelled with 0%ABV or the verbal descriptors Low or Super Low, whereas men, women, and those aged above 18 were perceived as the target groups for products labelled with higher %ABV. Participants also rated the products labelled with 0%ABV or the verbal descriptors Low or Super Low as targeting consumption on weekday lunches, whereas products labelled with higher %ABV were rated as targeting dinner/evening occasions, including parties, holidays and celebrations.
	Lachenmeier, D. W., Pflaum, T., Nieborowsky, A., Mayer, S., & Rehm, J. (2016). Alcohol-free spirits as novel alcohol placebo - A viable approach to	To test the hypothesis that consumers are not able to differentiate between alcohol free	Spirits	Editorial<br><br>ISO 4120 sensory analysis	Almost everyone in the taste trials was not only able to distinguish the two samples from each other, but also to correctly judge about the alcoholic strength of the beverage. The so-

	reduce alcohol-related harms? International Journal of Drug Policy, 32, 1-2. doi:10.1016/j.drugpo.2016.04.001	and alcoholic spirits in blind tasting			called "alcohol placebos" can be quite easily distinguished from the real alcohol.
	Saliba, A. J., Ovington, L. A., & Moran, C. C. (2013). Consumer demand for low-alcohol wine in an Australian sample. *International Journal of Wine Research*, *5*, 1-8.	To inform wine producers and marketers of those in the population who are interested in low-alcohol wine by describing the results of an Australian survey	Wine<br><br>Focus of the study	Online survey of adult wine consumers (N=851) on their purchasing and consumption of wine, demographics, knowledge, and reasons for consuming wine	The majority of respondents considered "low-alcohol wine" to contain around 3%–8% alcohol. Results indicated that those most likely to purchase low-alcohol wine were female and those who drink wine with food. Those who drank wine more frequently showed interest in wine sold in known-dose quantities, such as one standard drink. Reasons for preferring a low-alcohol wine included driving after drinking, to lessen the adverse effects of alcohol, and to consume more without the effects of a higher-alcohol wine. Finally, results pointed to the importance of taste as a driver of consumption.
**Policy drivers**					

**Marketing**	Vasiljevic, M., Coulter, L., Petticrew, M., & Marteau, T. M. (2018). Marketing messages accompanying online selling of low/er and regular strength wine and beer products in the UK: a content analysis. Bmc Public Health, 18. doi:10.1186/s12889-018-5040-6	To compare the main marketing messages conveyed by retailers and producers for low/er andregular strength wine and beer products.	Low alcohol products: (i.e., less than 1.2% ABV including de-alcoholised products)<br><br>Lower alcohol products: (i.e. above 1.2% ABV but less than the rate at which duty rises - 8.5% ABV for wine and 2.8% ABV for beer).<br><br>Regular strength: wines above 8.5% ABV and beers above 2.8% ABV.	Content analysis of the marketing messages stated (in text) or depicted (in image) for low/er and regular strength wines and beers sold online on the websites of the four main UK retailers (Tesco, ASDA, Sainsbury's, and Morrisons), and the producers of these products between February–March 2016.	Four themes were identified: (a) suggested occasions for consumption, (b) health-related associations, (c)alcohol content, and (d) taste.<br><br>Compared with regular strength products, low/er strength equivalents were more often marketed in association with occasions deemed to be suitable for their consumption including lunchtimes (wine), outdoor events/barbeques (beer) and on sport/fitness occasions (beer)<br><br>Compared with regular strength wines and beers, low/er strength equivalents were more frequently marketed with images or text associated with health. These included images of fruit and the provision of their energy (calorie) content.<br><br>Low/er strength products were also more often marketed with information about their alcohol content.<br><br>There were few differences in the marketing messages regarding taste<br><br>Low/er strength wines and beers appear to be marketed not as substitutes for higher strength products but as ones that can be consumed on additional occasions with an added implication of healthiness.

**Labeling**	Vasiljevic, M., Couturier, D. L., & Marteau, T. M. (2018). Impact of low alcohol verbal descriptors on perceived strength: An experimental study. British journal of health psychology, 23(1), 38-67. doi:10.1111/bjhp.12273	To examine consumers' perceptions of strength (% ABV) and appeal of alcohol products using low or high alcohol verbal descriptors	Focus of the study	Within-subjects experimental study in which participants rated the strength and appeal of 18 terms denoting low (nine terms), high (eight terms) and regular (one term) strengths for either (1) wine or (2) beer according to drinking preference<br><br>Adults (N=1600) (796 wine and 804 beer drinkers) sampled from a nationally representative UK panel.	Low, Lower, Light, Lighter, and Reduced formed a cluster and were rated as denoting lower strength products than Regular, but higher strength than the cluster with intensifiers consisting of Extra Low, Super Low, Extra Light, and Super Light. Similar clustering in perceived strength was observed amongst the high verbal descriptors.<br><br>Regular was the most appealing strength descriptor, with the low and high verbal descriptors using intensifiers rated least appealing.<br><br>Seventeen of 18 verbal descriptors for lower strength products were perceived as denoting products far higher in strength than the currently legislated cap of 1.2% ABV for low alcohol products
**Labeling**	Vasiljevic, M., Couturier, D. L., & Marteau, T. M. (2018). Impact on Product Appeal of Labeling Wine and Beer With (a) Lower Strength Alcohol Verbal Descriptors and (b) Percent Alcohol by Volume (%ABV): An Experimental Study. Psychology of Addictive Behaviors, 32(7), 779-791. doi:10.1037/adb0000376	To assess the impact of labeling wine and beer with different verbal descriptors denoting lower strength, with and without percent alcohol by volume (%ABV), on product appeal and understanding of strength.	Focus of the study	(N=3390) adult regular drinkers were randomized to 1 of 18 groups with 1 of 3 levels of verbal descriptor (Low vs. Super Low vs. No verbal descriptor) and 6 levels of % ABV (5 levels varying for wine and beer, and no level given).	Products with verbal descriptors denoting lower strength (Low and Super Low) had lower appeal than Regular strength products. Appeal decreased as %ABV decreased. Understanding of strength was generally high across the various drinks with majority of participants correctly identifying or erring on the side of caution when estimating the units and calories in a given drink, appropriateness for consumption by children, and drinking within the driving limit
	Vasiljevic, M., Couturier, D. L., Frings, D., Moss, A. C., Albery, I. P., & Marteau, T. M. (2018). Impact of Lower Strength Alcohol Labeling on Consumption: A Randomized Controlled Trial. Health Psychology, 37(7), 658-667. doi:10.1037/hea0000622	To test the hypothesis that labeling wine and beer as lower in alcohol increases their consumption	Low: 8% ABV in wine and 3% ABV in beer.<br><br>Super Low: 4% ABV in wine and 1% ABV in beer.<br><br>Regular: average % ABV available in the United Kingdom (12.9% for wine and 4.2% for beer), with no verbal descriptor of strength	Weekly wine and beer drinkers (N=264) sampled from a representative panel of the general population of England were randomized to one of three groups to taste test drinks in a bar-laboratory varying only in the label displayed; Group 1: verbal descriptor Super Low combined with 4% alcohol by volume (ABV) for wine/1%	The total amount of drink consumed increased as the label on the drink denoted successively lower alcohol strength. Group contrasts showed significant differences between those offered drinks labelled as Super Low compared with Regular, but not between Low and Regular.

			(which is how they are presented for sale).	ABV for beer; Group 2: verbal descriptor Low combined with 8% ABV for wine/3% ABV for beer; Group 3: no verbal descriptors of strength (Regular). Primary outcome was total volume (ml) of drink consumed.	
**Price**	Llopis, E. J., O'Donnell, A., & Anderson, P. (2021). Impact of price promotion, price, and minimum unit price on household purchases of low and no alcohol beers and ciders: Descriptive analyses and interrupted time series analysis of purchase data from 70, 303 British households, 2015-2018 and first half of 2020. Soc Sci Med, 270, 113690. doi:10.1016/j.socscimed.2021.113690	To estimate the potential impact of price in shifting British household purchases from higher to lower strength beers and cider	Alcohol free: all products with an ABV =0.0%<br><br>Lower strength: all products with an ABV >0.0% and ≤3.5% (a standard definition of low alcohol beers and ciders, hereafter lower strength);<br><br>Higher strength: all products with an ABV >3.5%	Descriptive statistics and controlled interrupted time series analyses using Kantar Worldpanel's British household purchase data from 70,303 households during 2015–2018 and the first half of 2020	No and low-alcohol products were less likely to be on price promotion than higher strength products.<br><br>No and low-alcohol beers were cheaper per volume than higher strength beers; the reverse was the case for ciders.<br><br>With the exception of low strength ciders (which had very few purchases) a higher volume was purchased when the product was on price promotion than when not.<br><br>Again, with the exception of low strength ciders, the cheaper the cost, the greater the volume of purchase, more so when the product was on price promotion.<br><br>The introduction of minimum unit price in Scotland (when controlling for changes in Northern England) and in Wales (when controlling for changes in Western England) shifted purchases from higher to lower strength products, more so for ciders than beers.<br><br>In relative terms, the alcohol by volume of beer dropped by 2% and of cider by 7%. Changes did not differ by household income or the age of the main shopper.
	Srivastava, P., McLaren, K. R., Wohlgenant, M., & Zhao, X. Y. (2015). Disaggregated econometric estimation of consumer demand response by alcoholic beverage types. Australian	To estimate price elasticities of demand for 12 disaggregated alcoholic beverages in Australia: premium beer, full strength beer, low alcohol beer and mid-strength	Low alcohol beer: 2.6% ABV<br><br>Mid strength beer: 3.4% ABV	The system of demand equations is estimated with Nielsen's data using a semiflexible Almost Ideal Demand System model in	Cross-price elasticities suggest that beverages most linked with negative externalities, namely full strength beer, dark RTD and dark spirits, may need to be taxed jointly. Any proposed tax increase to cask wine may also

	Journal of Agricultural and Resource Economics, 59(3), 412-432. doi:10.1111/1467-8489.12095	beer; red bottled wine, white bottled wine, sparkling wine, cask wine, dark and light ready-to-drink (RTD); and dark and light spirits.		order to impose negative semi-definiteness on the demand parameters	result in consumers shifting demand to more undesirable beverages.
	Vandenberg, B., Jiang, H., & Livingston, M. (2019). Effects of changes to the taxation of beer on alcohol consumption and government revenue in Australia. International Journal of Drug Policy, 70, 1-7. doi:10.1016/j.drugpo.2019.04.012	To examine the effect of the different tax policies on alcohol consumption and government revenue in Australian context	Low strength < 3.0% ABV<br><br>Mid strength 3.0-3.5% ABV<br><br>High strength > 3.5% ABV	We undertake time series analysis over 1989–2016 to examine the effect of beer tax policies in two sub-periods (before/after 2000/01) on category-level beer consumption per capita and government revenue. We alsotest if the policy changes in 2000/01 had immediate or long-term effects on total (all beer category) consumptionover 1989–2016. Data includes monthly domestic beer sales volumes by category (in litres of alcohol), monthlygovernment revenue from beer tax (AUD$), and inflation-adjusted tax rates (AUD$ per litre of alcohol).	Un 2000/01, varying nominal rates of tax were introduced for beer products according to three alcohol content levels (low-/mid-/high-strength) and container type (on-/off-pre-mises).<br><br>Before 2000/01, the single nominal tax rate had a significant positive effect on revenue, but no significant effect on consumption.<br><br>After 2000/01, the relatively higher nominal tax rates for two beer categories (mid- and high-strength off-premises) had a significant negative effect on their consumption, and a significant negative effect on revenue in one category (mid-strength off-premises). However, across the full period examined (1989–2016), the level and slope of total beer consumption was not significantly affected by the tax policy changes in 2000/01.
**Reviews - general**					

**Production**	Baiano, A. (2020). Craft beer: An overview. Compr Rev Food Sci Food Saf. doi:10.1111/1541-4337.12693	The purpose of the work was to provide an overview on craft beer, including details and issues concerning history and legal definition market, fiscal policy, innovation, safety, healthiness, consumer profile, and sustainability.		Review	As gluten-free beers, low alcoholic or alcohol-free beers also represent a niche in the niche of craft beers, and as a consequence of increased health interest, some small brewers started producing low and non-alcoholic beer. Strategies to reduce the alcohol content include vacuum-distillation, reverse osmosis (which involves very expensive equipment), interrupted fermentation (a possible defect is a sugary, unbalanced malt taste), and a so-called "craft" version (involving the use of mash bill with a wide variety of ingredients able to give the beer more "body," and the use of low alcohol yeast strains, generally non-Saccharomyces strains that cannot ferment maltose or maltotriose)<br><br>The current trend is to produce "isotonic" claimed beers by combining low-alcoholic contents (<0.5% v/v) with iso-tonic properties so creating new beverages with characteristics similar to sport drinks, but also rich in antioxidant compounds and having an appropriate osmolality. (according to the European Food Safety Authority, the osmolality of isotonic beverages is in the 270 to 330 mOsmol/kg range).

**Production**	Hagemann, M. H., Bogner, K., Marchioni, E., & Braun, S. (2016). Chances for dry-hopped non-alcoholic beverages? Part 1: Concept and market prospects. Brewing Science, 69(7-8), 50-55. Retrieved from <Go to ISI>://WOS:000413980700002	To estimate the market potential for dry-hopped non-alcoholic beverages in the European and Northern American markets considering hop and (craft) beer production, consumption of alcoholic and non-alcoholic beverages as well as health properties for marketing purposes.	Beers that contain less than 0.1 vol%, which are still rare, typical AFB, often with a rest alcohol content of 0.5 vol% or less,<br>low alcohol beers with less than 3 vol% of alcohol Following EU regulations, it is only necessary to explicitly state the alcohol content if a food product contains more than 1.2 vol% (EP No. 1169/2011). Within Europe the threshold is similar; between 0.5 vol% (e.g. UK, Germany, Switzerland), 1 vol% (Portugal, Spain)	Review	The markets for craft beer, for alcohol-free beer and for functional drinks are rapidly growing, thus it seems that the consumer is prepared to a new product type with a bitter but aromatic and complex taste. This could be achieved by dry-hopping as this technique is easy to introduce and the resulting product addresses the consumers demand for alcohol-free beverages and distinct taste of craft beers. Challenges are the sensory properties due to the low alcohol concentration and a resulting lack of “body”. Rich hop flavors may cover this to a sufficient extent.

**Perception and preferences**	Myles, C. C., Goff, P. D., Wiley, D., & Savelyev, A. (2020). Low Gravity on the Rise: A Sociocultural Examination of Low Alcohol Beer in the United States. In *The Geography of Beer* (pp. 87-100). Springer, Cham.	Using examples from the United States (US), this paper explores the increasing demand for low- to no-alcohol beer, surveying the sociocultural motivations for, and contemporary patterns of, the production and consumption of “near” or “session” beer.	Not clearly defined – in the empirical part: less than or equal to 5% ABV for styles that are traditionally known as low alcohol, and settled on the following styles to define low alcohol beer: session beer (ale, IPA), gose, berliner, and kolsch (80% were less than or equal to 5% ABV) , lager, saison, and pilsner.	Based on a review of scholarly and popular literature, social media mentions, and regulatory restriction	the period of time surveyed within the US). The map in Fig. 4 captures a few broad regions that appear to have a higher rate of low alcohol beer mentions compared to the national average. Hotspots exist10 in Utah, Colorado, Ari- zona, Nebraska, Arkansas, South Carolina, and on the Washington-Idaho border.<br><br>Factors driving the growing demand for low gravity beers, such as: dietary and health preferences, regulation and taxation, mounting awareness and acknowledgment of the dangers of drinking and driving, and an overall decrease in social stigma associated with non-alcoholic beverages.<br><br>Twitter mentions analysis: The low alcohol beer mentions amount to about 7.52% of all beer mentions (for the period of time surveyed within the US). Broad regions that appear to have a higher rate of low alcohol beer mentions compared to the national average are Utah, Colorado, Ari- zona, Nebraska, Arkansas, South Carolina, and on the Washington-Idaho border.

**Health**	Rehm, J., Lachenmeier, D. W., Llopis, E. J., Imtiaz, S., & Anderson, P. (2016). Evidence of reducing ethanol content in beverages to reduce harmful use of alcohol. Lancet Gastroenterology & Hepatology, 1(1), 78-83. doi:10.1016/s2468-1253(16)30013-9	To examine evidence base of reduction of alcoholic strength of beer as mean to reduce harmful use of alcohol.			three potential mechanisms for how reduction of alcoholic strength could affect harmful use of alcohol:<br>- by current drinkers replacing standard alcoholic beverages with similar beverages of lower alcoholic strength, without increasing the quantity of liquid consumed;<br>- by current drinkers switching to no alcohol alternatives for part of the time, thereby reducing their average amount of ethanol consumed;<br>- and by initiating alcohol use in current abstainers.<br><br>The first mechanism seems to be the most promising to potentially reduce harm, but much will depend on actual implementation, and only an independent assessment will be able to identify effects on harmful drinking. The potential of alcoholic strength reduction is independent of initiation by law or by self-initiative of the industry
